# HILAMA: High-dimensional multi-omics mediation analysis with latent confounding

**DOI:** 10.1186/s12874-025-02686-z

**Published:** 2025-10-24

**Authors:** Xinbo Wang, Junyuan Liu, Sheng’en Shawn Hu, Zhonghua Liu, Hui Lu, Lin Liu, and for the Alzheimer’s Disease Neuroimaging Initiative

**Affiliations:** 1 Department of Bioinformatics and Biostatistics, School of Life Sciences and Biotechnology, Shanghai Jiao Tong University, Shanghai, 200240 China; 2 SJTU-Yale Joint Center for Biostatistics and Data Science, Technical Center for Digital Medicine, National Center for Translational Medicine, Shanghai Jiao Tong University, Shanghai, 200240 China; 3 School of Mathematical Sciences, Shanghai Jiao Tong University, Shanghai, China; 4 Department of Genome Sciences, University of Virginia, Charlottesville, VA USA; 5 Department of Biostatistics, Columbia University, New York, NY USA; 6 Shanghai Engineering Research Center for Big Data in Pediatric Precision Medicine, Center for Biomedical Informatics, Shanghai Children’s Hospital, School of Medicine, Shanghai, China; 7 Institute of Natural Sciences, MOE-LSC, CMA-Shanghai, Shanghai Jiao Tong University, Shanghai, China

**Keywords:** High-dimensional mediation analysis, Latent confounding, False discovery rate control, Multi-omics, Alzheimer’s disease

## Abstract

**Background:**

The increasingly available multi-omics datasets have posed both new opportunities and challenges to the development of quantitative methods for discovering novel mechanisms in biomedical research. One natural approach to analyzing such datasets is mediation analysis originated from the causal inference literature. Mediation analysis can help unravel the mechanisms through which exposure(s) exert the effect on outcome(s). However, existing methods fail to consider the case where (1) both exposures and mediators are potentially high-dimensional and (2) it is very likely that some important confounding variables are unmeasured or latent; both issues are quite common in practice. To the best of our knowledge, however, no methods have been developed to address these challenges with statistical guarantees.

**Methods:**

In this article, we propose a new method for HIgh-dimensional LAtent-confounding Mediation Analysis (HILAMA) that considers both high-dimensional exposures and mediators, as well as the possible existence of latent confounding variables. HILAMA employs the *Decorrelating* & *Debiasing* method to estimate the individual effects of exposures and mediators on the outcome. A column-wise regression strategy with parallel computing is considered to efficiently estimate the exposure-mediator effect matrix. HILAMA then applies the *MinScreen* procedure to eliminate non-significant pairs, and the Joint-Significance Testing (JST) method to compute *p*-values for the retained pairs, controlling the False Discovery Rate (FDR) using the Benjamini-Hochberg (BH) procedure.

**Results:**

The proposed method is evaluated through extensive simulation experiments, demonstrating its improved stability in FDR control and superior power in finite sample size compared to existing competitive methods. Furthermore, our method is applied to the proteomics-radiomics data from ADNI, identifying some key proteins and brain regions related to Alzheimer’s disease. These empirical results demonstrate that HILAMA can effectively control FDR and provide valid statistical inference for high dimensional mediation analysis with latent confounding variables under certain assumptions.

**Conclusions:**

HILAMA can effectively control FDR and provide valid statistical inference for high dimensional mediation analysis with latent confounding variables under certain assumptions.

**Supplementary Information:**

The online version contains supplementary material available at 10.1186/s12874-025-02686-z.

## Background

The emergence of modern biotechnologies, such as high-throughput omics and multimodal neuroimaging, has led to the rapid accumulation of omics data at various levels, often including information on genomics, epigenomics, transcriptomics, proteomics, radiomics, and clinical records. It then becomes possible to thoroughly study complex diseases such as cancer and Alzheimer’s disease by integrating information from various scales [1, 2]. For example, large collaborative consortia such as Alzheimer’s Disease Neuroimaging Initiative (ADNI) have collected information across all the levels mentioned above to help unravel the causal mechanisms of Alzheimer’s disease [3]. It is thus urgently needed to develop rigorous statistical methods for analyzing such datasets to reliably dissect the causal mechanisms [4–6].

Such a problem falls into the category of causal mediation analysis [7, 8], which can help disentangle the intermediate mechanisms between cause-effect pairs from observational datasets [9–13]. The classical mediation analysis can be traced back to 1930 s [14], and then reverberated in early 1980 s based on regression techniques [8]. Over the following decades, a vast literature has been engendered to put mediation analysis on a more rigorous ground both mathematically and conceptually [15–18]. We refer interested readers to VanderWeele [7] for a textbook-level introduction.

However, methods for mediation analyses with a single or a few exposures and/or mediators often cannot be directly scaled to address high-dimensional omics data. For instance, traditional hypothesis testing methods, such as Joint Significance Test (JST) [19], Sobel’s method [20], and bootstrap method [21], tend to be overly conservative particularly in genome-wide epigenetic studies [22, 23].

In response to the above challenge, recent years have seen a surge in the development of new methods for high-dimensional mediation analysis. These methods aim to explore the biological mechanisms derived from multi-omics data [24, 25]. For instance, some have focused on epigenetic studies or neuroimaging studies with high-dimensional mediators and a continuous outcome [26–30]. They have primarily employed (debiased/de-sparsified) penalized linear regression and multiple testing procedures to formulate their methods. Derkach et al. [31] have tackled a similar problem by considering multiple latent variables as mediators that influence both the high dimensional biomarkers and the outcome [31]. Furthermore, the continuous outcome setting has also been extended in a few recent works to survival outcome settings, alongside high-dimensional mediators [32–34]. Additionally, mediation analysis with high-dimensional continuous mediators has also been further developed to handle compositional microbiome data [35–38]. In a similar vein, Shao et al. [39] investigated high-dimensional exposures with a single mediator in an epigenetic study, utilizing a linear mixed-effect model [39]. All the above studies have contributed to the growing literature on exploring complex biological relationships.

Specifically for neuroimaging data analysis, Zhao et al. [40] recently developed a novel method that deals with multivariate-mediator and multivariate-outcome [40]. However, there have been limited works studying both multivariate exposures and mediators. Zhang [41] considered high-dimensional exposures and mediators through two different procedures; however, they require the mediators to be jointly independent and mainly focus on the mediator selection problem [41]. Zhao et al. [42] developed a novel penalized principal component regression method that replaces the exposures with their principal components in a lower dimension [42]. This approach, however, lacks direct causal interpretation when the exposures are scientifically meaningful. More importantly, most high-dimensional mediation analysis approaches make the untestable assumption of no latent confounding, which is highly problematic in multi-omics biological studies due to the prevalence of non-randomized study designs. If hidden confounding cannot be ruled out, these methods can be biased due to spurious correlations and result in an inflated False Discover Rate (FDR) [43]. Recently, several works have been devoted to tackle this problem in the simpler high-dimensional linear models, which can be viewed equivalently as estimating the total causal effects of the exposures under the Latent Structural Equation Modeling (LSEM) framework [44–47]. Specifically, Sun et al. [48] are the first to address the large-scale hypothesis testing problem in the high-dimensional confounded linear model by proposing the *Decorrelating* & *Debiasing* estimator [48]. They achieve FDR control under finite sample sizes by introducing a decorrelating transformation prior to the debiasing step.

Inspired by the aforementioned works on high-dimensional linear regression with latent confounding [44–48], we propose a novel method called HILAMA, which stands for HIgh-dimensional LAtent-confounding Mediation Analysis. In contrast to the focus of Sun et al. [48] on hypothesis testing in confounded linear models, HILAMA extends this framework to mediation analysis, thereby enabling a comprehensive examination of causal pathways in high-dimensional multi-omics contexts. HILAMA addresses two critical challenges in applying mediation analysis (or any causal inference method) to multi-omics studies: (1) accommodating both high-dimensional exposures and mediators, and (2) effectively handling latent confounding. In contrast to competing methods [8, 26, 27, 42, 49], our method maintains control of FDR at the nominal level for multiple direct/indirect effect testing, even in the presence of latent confounders. Now, we briefly sketch the essential components of our method. First, we employ the *Decorrelating* & *Debiasing* method [48] to obtain *p*-values for each individual effects of the exposures and mediators on the outcome. Second, to estimate the effect matrix of exposures on mediators, we employ a column-wise regression strategy, again incorporating the *Decorrelating* & *Debiasing* method [48]. To handle large and high-dimensional datasets, we utilize parallel computing in this step. Third, we apply the *MinScreen* procedure to eliminate non-rejected hypotheses [50], retaining only the *K* most significant pairs for the final stage of multiple testing. Lastly, we compute *p*-values for all *K* pairs using the JST method [19], employing a data-dependent threshold determined by the Benjamini-Hochberg (BH) procedure [51] to maintain FDR at the nominal level $\alpha$. We conduct extensive simulations to evaluate the finite sample performance of our method, demonstrating effective FDR controls across various sample sizes, compared to most of the other competing methods in the presence of hidden confounding. Finally, we apply HILAMA to a proteomics-radiomics dataset from the ADNI database (adni.loni.usc.edu) and identify key proteins and brain regions associated with learning, memory, and recognition impairments in Alzheimer’s disease and cognitive impairment.

The rest of this article is organized as follows. We first introduce the proposed model HILAMA, under the Linear Structural Equation Models with high-dimensional exposures, high-dimensional mediators, continuous outcome, and latent confounding. In the next part, we evaluate the FDR and Power performance of HILAMA across a wide range of simulations and analyze the indirect effects of high dimensional proteins on cognitive function through high dimensional imaging information of brain regions as mediators, using a proteomics-radiomics data of Alzheimer’s disease from ADNI. Finally, we conclude the paper with a discussion on the limits and other possible extensions.

## Methods

### Notations and HIgh-dimensional LAtent-confounding Mediation Analysis (HILAMA)

**Fig. 1:**
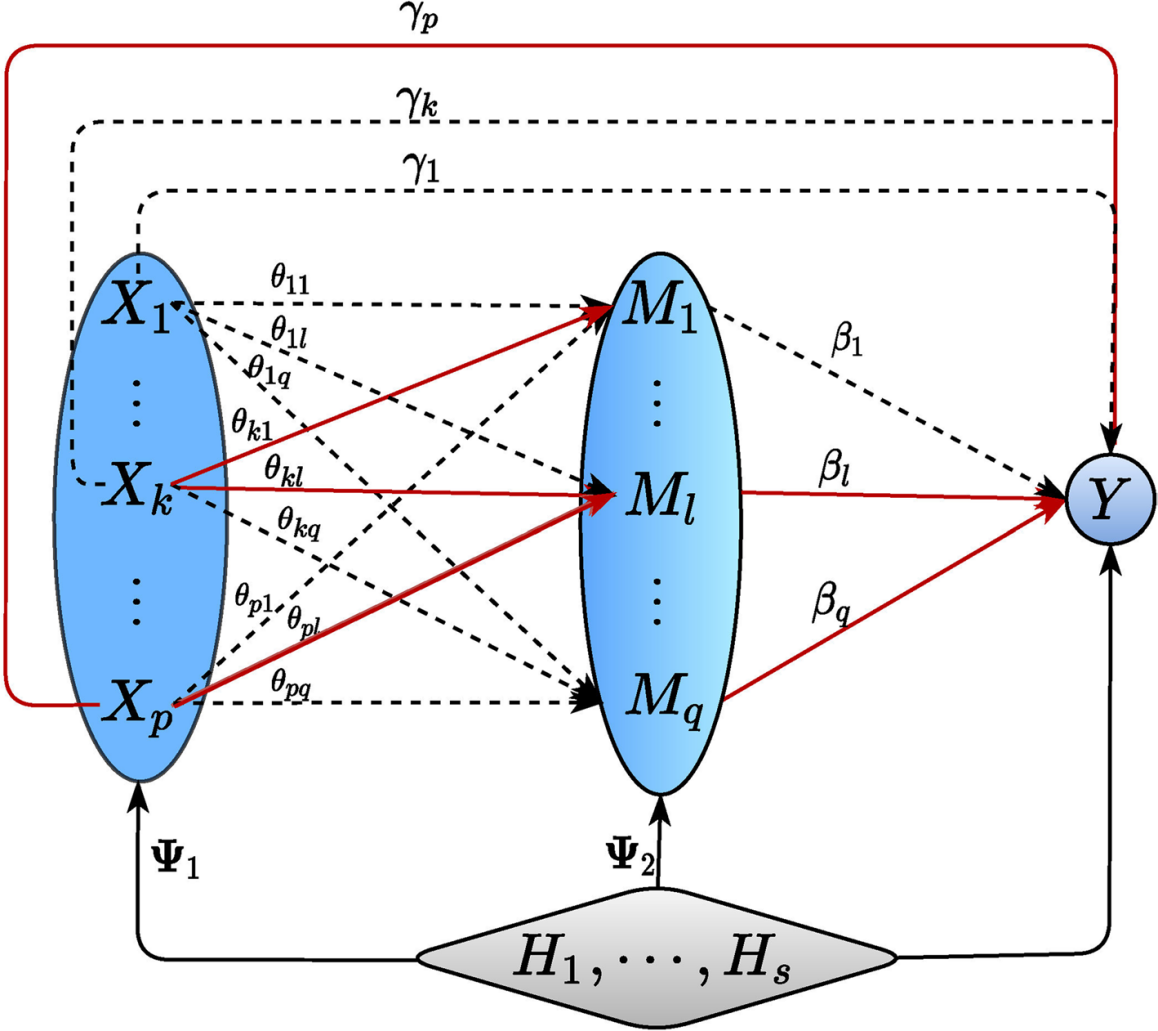
Causal Diagram considered in this paper. $\textbf{X} = (X_1, \cdots , X_p)^\top$: exposures of dimension *p* (protein expression data in our real-data applications); $\textbf{M} = (M_1, \cdots , M_q)^\top$: mediators of dimension *q* (imaging data in our real-data applications); *Y*: a real-valued outcome (clinical outcome or phenome data in our real-data applications); $\varvec{H} = (H_1, \cdots , H_s)^\top$: latent/unmeasured confounders of dimension *s* (e.g. mis-measured clinical data, epigenetic information, etc.). Here, we omit the observed baseline covariates $\textbf{Z}$ for the sake of presentation convenience. Solid (red) lines indicate non-null effects, while dotted lines indicate null effects

To describe the HILAMA methodology, we first need to briefly review mediation analyses. Mediation analyses are frequently utilized to disentangle the underlying causal mechanism between two sets of variables, the exposures and outcomes, exerted by a third set of variables, the mediators. The overall causal effects can be decomposed into direct effects from exposures to outcomes, bypassing mediators, and indirect effects via mediators. To be more precise, we ground our discussion under the Linear Structural Equation (LSE) framework, that models the causal mechanisms among *p*-dimensional exposures $\textbf{X}_i=(X_{i1},\cdots ,X_{ip})^\top \in \mathbb {R}^p$, *q*-dimensional mediators $\textbf{M}_i=(M_{i1},\cdots ,M_{iq})^\top \in \mathbb {R}^q$, *r*-dimensional baseline covariates $\textbf{Z}_i=(Z_{i1},\cdots ,Z_{ir})\in \mathbb {R}^r$, a scalar outcome $Y_i\in \mathbb {R}$, and latent confounders $\textbf{H}_i=(H_{i1},\cdots ,H_{is})^\top \in \mathbb {R}^s$ (e.g., batch effects, disease subtypes, and lifestyle factors) as follows:

1 $$\begin{aligned} Y_i & =\textbf{X}_{i}^\top \varvec{\gamma }+\textbf{M}_{i}^\top \varvec{\beta } + \textbf{Z}_i^\top \varvec{\phi } +\textbf{H}_{i}^\top \varvec{\psi } + \epsilon _i ,\end{aligned}$$

2 $$\begin{aligned} \textbf{M}_{i} & = \varvec{\Theta }^\top \textbf{X}_{i} + \varvec{\Phi }_2^\top \textbf{Z}_i+ \varvec{\Psi }_2^\top \textbf{H}_i +\textbf{E}_{M_i} , \end{aligned}$$

where $\epsilon _i \text { and } \textbf{E}_{Mi}$ are the noise terms that are independent of $\textbf{X}_i,\textbf{M}_{i},\textbf{Z}_i$ and $\textbf{H}_i$. In the outcome model (1), $\varvec{\gamma }=(\gamma _1,\cdots ,\gamma _p)^\top \in \mathbb {R}^{p}$ is the direct effect vector of the exposures $\textbf{X}_{i}$ on the outcome $Y_i$, and $\varvec{\beta }=(\beta _1,\cdots ,\beta _q)^\top \in \mathbb {R}^{q}$ represents the effect vector of the mediators $\textbf{M}_i$ to the outcome $Y_i$ after adjusting for the baseline covariates $\textbf{Z}_i$, and the latent confounders $\textbf{H}_i$. $\varvec{\psi }\in \mathbb {R}^{s}$ is the parameter vector that relates latent confounders $\textbf{H}_i$ to the outcome $Y_i$. Here, we allow *p* and *q* to be larger than the sample size *n*, while *s* is small compared to the *p* and *q*. The primary objective of our study is to identify the active direct/indirect effects ($\gamma _k, \theta _{kl}\beta _{l}, k\in [p], l\in [q]$) from *p*/*pq* possible paths, as shown in Fig. 1. Here, [*p*] represents the set $\{1,2,\cdots ,p\}$.

In the mediator model (2), the matrix $\varvec{\Theta }=(\varvec{\theta }_1,\cdots ,\varvec{\theta }_q)=(\theta _{kl})\in \mathbb {R}^{p\times q}$ represents the regression coefficients of exposures on mediators and $\theta _{kl}$ represents the effect of exposure $X_{ik}$ on mediator $M_{il}$ after adjusting for the effect of latent confounders $\textbf{H}_i$. $\varvec{\Psi }_2 \in \mathbb {R}^{s\times q}$ can be interpreted as the confounding effect of the latent confounders $\textbf{H}_i$ on mediators $\textbf{M}_i$. The mediation model (1) – (2) adopted here is similar to those proposed in [42, 49], which were among the first works to consider both multivariate exposures and mediators. However, we incorporate the latent confounders into our high-dimensional mediation analysis, which is a novel approach in the field. Furthermore, unlike the approach used in [42], our mediation analysis is directly based on $\textbf{X}_i$, instead of a transformation $\tilde{\textbf{X}}_i$ of the original vector $\textbf{X}_i$.

As mentioned, the causal parameters of interest in mediation analyses are mainly the (average) natural direct and indirect effects. When the ignorability assumption (explained in Supplementary Information) holds [15, 16], the natural direct effect of exposure $X_k$ on outcome, denoted by $\textrm{NDE}_k$, and the natural indirect effect of exposure $X_k$ on outcome, denoted by $\textrm{NIE}_k$, can be expressed as

$$\begin{aligned} \textrm{NDE}_k (x_k, x_k^*) & = \gamma _k (x_k-x_k^{*}) , \quad k\in [p] ,\\ \textrm{NIE}_k (x_k, x_k^*) & = \sum _{l=1}^{q} \theta _{kl}\beta _l (x_k - x_k^{*} )\\ & := \sum _{l=1}^{q} \text {NIE}_{kl} (x_k, x_k^*), \quad k\in [p]. \end{aligned}$$

When $x_{k}$ and $x_{k}^{*}$ defer by one unit, $\textrm{NDE}_{k} (1) = \gamma _{k}$, the regression coefficient between $\textbf{X}$ and *Y* in model (1), and $\textrm{NIE}_{kl} (1) = \theta _{kl} \beta _{l}$, the product of the regression coefficient between $\textbf{X}_{k}$ and $\textbf{M}_{l}$ in model (2) and the regression coefficient between $\textbf{M}_{l}$ and *Y* in model (1). For their derivation, see Supplementary Information.

However, in the presence of latent confounders (i.e. $\text {dim}(\textbf{H})> 0$), neither NDEs nor NIEs are identifiable without making additional assumptions, which are generally based by domain-specific knowledge. To identify the true parameter $\varvec{\gamma }, \varvec{\beta } \text { and } \varvec{\theta }_l$ in the confounded linear model (1) and (2), it is necessary to make additional assumptions among the observed variables $(\textbf{X}_i,\textbf{M}_i)$ and the latent confounders $\textbf{H}_i$. Based on the aforementioned works [45, 46, 48, 52], a factor model is specified to characterize the relation between $\textbf{X}_i$ and $(\textbf{Z}_i^\top ,\textbf{H}_i^\top )^\top$:

3 $$\begin{aligned} \textbf{X}_i = \varvec{\Phi }_1^\top \textbf{Z}_i + \varvec{\Psi }_1^\top \textbf{H}_i + \textbf{E}_{X,i}, \end{aligned}$$

where $\textbf{Cov}((\textbf{Z}_i^\top ,\textbf{H}_i^\top )^\top ,\textbf{E}_{X,i})=0$ and the random variable $\textbf{E}_{X,i}\in \mathbb {R}^{p}$ represents the unconfounded components of $\textbf{X}_i$. Moreover, to accurately identify the true signals and effectively remove the confounding effects, we impose a spiked singular value condition on the covariance between the exposures and mediators, as shown in Supplementary Information. Specifically, we require $\lambda _s(\varvec{\Psi }) = O(\sqrt{p+q})$, where $\varvec{\Psi }= \left( \varvec{\Psi }_{1}, \varvec{\Psi }_2\right) \in \mathbb {R}^{s\times (p+q)}$ and $\lambda _{s}$ denotes the *s*-th largest singular values [48]. Our approach is particularly effective in scenarios where the confounding effect is dense, i.e., many observed variables in $\textbf{X} \in \mathbb {R}^{p}$ and $\textbf{M} \in \mathbb {R}^{q}$ are simultaneously influenced by the latent confounders $\textbf{H} \in \mathbb {R}^{s}$.

Our goal is to identify the path-specific indirect effect $\theta _{kl}\beta _l$ (corresponding to the path $X_k \rightarrow M_l \rightarrow Y$) from the total $p\cdot q$ possible paths which corresponds to the following multiple hypothesis testing problem:

4 $$\begin{aligned} H_{0,kl}: \theta _{kl}\beta _l=0 \quad v.s. \quad H_{1,kl}:\theta _{kl}\beta _l\ne 0 ,\quad k\in [p], \quad l\in [q]. \end{aligned}$$

### HILAMA procedure

**Fig. 2:**
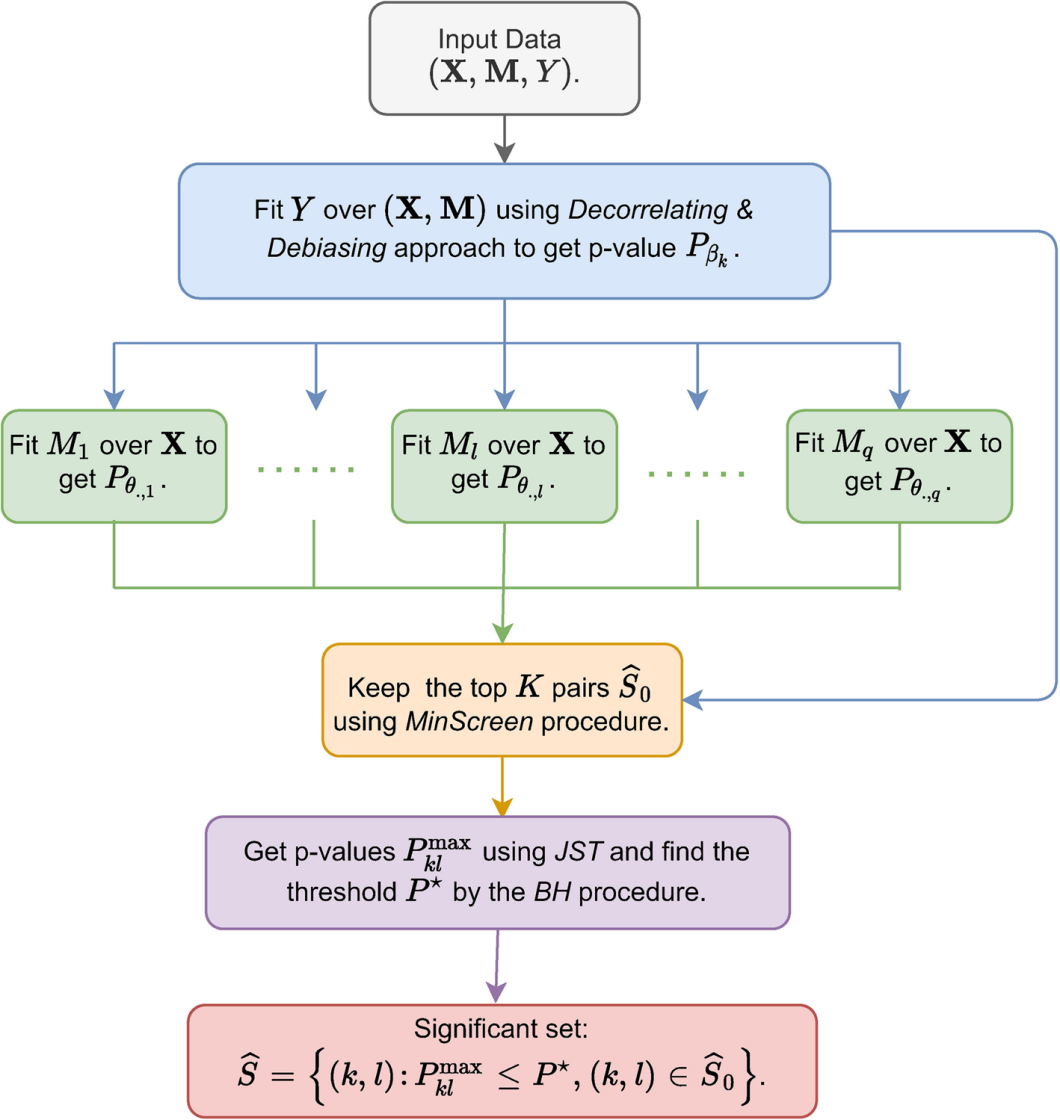
Flowchart of HILAMA. First, we regress outcome *Y* over mediators $\textbf{M}$ and exposures $\textbf{X}$ using the *Decorrelating* & *Debiasing* approach to obtain the debiased *p*-values for the parameter $\varvec{\beta }$. Second, we similarly perform parallel regression analyses of each mediator $M_l$ over the exposures $\textbf{X}$ to obtain debiased *p*-values for the parameter $\varvec{\Theta }$. Third, we employ the *MinScreen* procedure to select a subset of *K* pairs for subsequent multiple testing. Finally, we calculate *p*-values for the mediation effect using the *JST* method and choose a *p*-value threshold using the *BH* procedure based on a pre-specified FDR level, where a set of pairs is considered significant if the *p*-value falls below this threshold

Here, we propose a novel framework called HILAMA to solve the hypothesis testing problem (4) for high-dimensional mediation analysis in the presence of hidden confounding. The framework identifies the true paths with nonzero indirect effects and controls the finite-sample FDR. It involves four major steps as illustrated below (as shown in Fig. 2).

First, for the outcome model in Eq. (1), we utilize the *Decorrelating* & *Debiasing* approach to carry out inference on the regression parameters $\varvec{\gamma }$ and $\varvec{\beta }$ [48]. Specifically, let $\textbf{Y} = (Y_1, \ldots , Y_n)^\top$, $\textbf{X}^\top = (\textbf{X}_1, \ldots , \textbf{X}_n)$, $\textbf{M}^\top = (\textbf{M}_1, \ldots , \textbf{M}_n)$, $\textbf{Z}^\top = (\textbf{Z}_1, \ldots , \textbf{Z}_n)$, and $\textbf{H}^\top = (\textbf{H}_1, \ldots , \textbf{H}_n)$. For simplicity, we first assume the absence of $\textbf{Z}$. To further avoid notation clutter, we concatenate the exposures and mediators into $\textbf{O} {:=} (\textbf{X}, \textbf{M}) \in \mathbb {R}^{n \times (p+q)}$ and their coefficients into $\varvec{\alpha } {:=} (\varvec{\gamma }^\top , \varvec{\beta }^\top )^\top \in \mathbb {R}^{(p+q)}$. Equations (1) – (3) can then be reduced to:

5 $$\begin{aligned} \textbf{Y} & = \textbf{O} \varvec{\alpha } + \textbf{H} \varvec{\psi } + \varvec{\epsilon }, \end{aligned}$$

6 $$\begin{aligned} \textbf{O} & = \textbf{H} \varvec{\Psi }_o + \textbf{E}_{o}, \end{aligned}$$

where $\varvec{\Psi }_o = (\varvec{\Psi }_1, \varvec{\Psi }_1 \varvec{\Theta } + \varvec{\Psi }_2), \textbf{E}_{o}=(\textbf{E}_X,\textbf{E}_X\varvec{\Theta }+\textbf{E}_M)$. Since $\textbf{H}\varvec{\Psi }_o = \textbf{H} \varvec{U}^\top \varvec{U}\varvec{\Psi }_o$ holds for any $s\times s$ orthogonal matrix $\varvec{U}$, following the factor analysis literature [52], we further impose–without loss of generality–the identifiability conditions: $\frac{1}{n}\varvec{H}^{\top }\varvec{H} = \varvec{I}_s$ and $\varvec{\Psi }_o^{\top }\varvec{\Psi }_o$ is diagonal. If we project $\textbf{H}$ onto the linear span of $\textbf{O}$, we have $\textbf{H} \varvec{\psi } = \textbf{O} \textbf{b} + (\textbf{H} \varvec{\psi } - \textbf{O} \textbf{b})$ for some $\textbf{b}$ such that $\text {Cov}(\textbf{O}, \textbf{H} \varvec{\psi } - \textbf{O} \textbf{b}) = \varvec{0}$. Consequently, the outcome model (5) can be represented as a linear model with a coefficient different from the truth $\varvec{\alpha }$ by a bias $\textbf{b}$:

7 $$\begin{aligned} \textbf{Y} = \textbf{O} (\varvec{\alpha } + \textbf{b}) + \varvec{\xi }, \quad \text {where } \varvec{\xi } = \textbf{H} \varvec{\psi } - \textbf{O} \textbf{b} + \varvec{\epsilon }. \end{aligned}$$

To mitigate the bias $\textbf{b}$ caused by latent confounders $\textbf{H}$, a decorrelating matrix $\textbf{F}_{\text {dc}} = \sum _{i=s+1}^{n} u_i u_i^\top$ is left-multiplied to Eqs. (5) and (6), with $\textbf{O} = \sum _{i=1}^{n} \lambda _i u_i v_i^\top$ representing the singular value decomposition (SVD) of the design matrix $\textbf{O}$, where $\lambda _1 \ge \lambda _2 \ge \cdots \ge \lambda _n$. This decorrelating operation turns the data generating model into:

8 $$\begin{aligned} \tilde{\textbf{Y}} & = \tilde{\textbf{O}} \varvec{\alpha } + \tilde{\textbf{O}} \textbf{b} + \tilde{\varvec{\xi }}, \end{aligned}$$

9 $$\begin{aligned} \tilde{\textbf{O}} & = \tilde{\textbf{H}} \tilde{\varvec{\Psi }}_o + \tilde{\textbf{E}}_o, \end{aligned}$$

where $\tilde{\cdot } = \varvec{F}_{\text {dc}} \cdot$. After the transformation, the confounding term $\tilde{\textbf{O}} \textbf{b}$ is reduced due to the removal of the top *s* largest singular values ($\tilde{\textbf{O}} = \sum _{i=s+1}^{n} \lambda _i u_i v_i^\top$). This also results in weaker correlations among the components of $\tilde{\textbf{O}}$.

#### Remark 1

We provide a heuristic explanation on why multiplying the structural Eqs. (5) and (6) by $F_\text {dc}$ reduces bias. To estimate the latent confounders $\textbf{H}$ and the loading matrix $\varvec{\Psi }_0$ in (6), we apply factor analysis to $\textbf{O}$, yielding:

$$\begin{aligned} \hat{\textbf{H}} = \sqrt{n} \left( u_1, \cdots , u_s \right) , \quad \hat{\varvec{\Psi }}_{0}^\top = \frac{1}{\sqrt{n}} \left( \lambda _1 v_1, \cdots , \lambda _s v_s \right) , \end{aligned}$$

where $\textbf{O} = \sum _{i=1}^{n} \lambda _i u_i v_i^\top$. The orthogonal projection matrix onto the complement of $\varvec{H}$ is then:

$$\begin{aligned} P_{\hat{H}}^{\perp } = I_n - P_{\hat{H}} = I_n - \hat{H} (\hat{H}^\top \hat{H})^{-1} \hat{H}^\top = \sum _{i=s+1}^{n} u_i u_i^\top , \end{aligned}$$

which equals $F_\text {dc}$. Left-multiplying by $F_\text {dc}$ projects the data onto the space orthogonal to $\varvec{H}$, thereby mitigating the confounding impact of $\varvec{H}$ on the observed data.

Based on the transformed data $(\tilde{\textbf{O}}, \tilde{\textbf{Y}})$ and the assumption that $\Vert \textbf{b}\Vert _2$ is of small order of magnitude for large covariates under dense confounding [46, 48], the *Decorrelating* & *Debiasing* estimator can be derived analogously to the debiased Lasso procedure [53]. This estimator is expressed as follows:

10 $$\begin{aligned} \hat{\varvec{\alpha }}_j=\check{\varvec{\alpha }}_j + \frac{\textbf{v}_j^\top \left( \tilde{\textbf{Y}}-\tilde{\textbf{O}}\check{\varvec{\alpha }}\right) }{\textbf{v}_j^\top \tilde{\textbf{O}}_{.,j}},\quad j=1,2\cdots ,(p+q) \end{aligned}$$

where initial estimation $\check{\varvec{\alpha }}$ can be obtained by regressing $\tilde{\textbf{Y}}$ on $\tilde{\textbf{O}}$ using Lasso and $\textbf{v}_j$ can be obtained as follows

11 $$\begin{aligned} \textbf{v}_j=\tilde{\textbf{O}}_{.,j} - \tilde{\textbf{O}}_{.,-j}\hat{\varvec{\zeta }}_j, \quad j=1,2,\cdots ,(p+q) \end{aligned}$$

where $\hat{\varvec{\zeta }}_j$ can be the Lasso estimator by regressing $\tilde{\textbf{O}}_{.,j}$ on $\tilde{\textbf{O}}_{.,-j}$.

When baseline covariates $\textbf{Z}$ are present, we initially project the observed data onto the orthogonal complement of the span of $\textbf{Z}$ to eliminate the influence of $\textbf{Z}$. Specifically, let the projection matrix be $\textbf{P}_z=\textbf{Z}(\textbf{Z}^\top \textbf{Z})^{-1}\textbf{Z}^\top$ and its orthogonal complement be $\textbf{P}_z^\perp =\textbf{I}_n-P_z$. By left-multiplying Eq. (12) with the projection matrix $\textbf{P}_z^\perp$,

12 $$\begin{aligned} \textbf{Y}=\textbf{X} \varvec{\gamma } + \textbf{M} \varvec{\beta } + \textbf{Z} \varvec{\phi } + \textbf{H}\varvec{\psi }+\varvec{\epsilon }, \end{aligned}$$

we can eliminate the effect of $\varvec{Z}$.

These estimators, after appropriate rescaling, asymptotically converge to centered Gaussian distributions: $\sqrt{n} (\hat{\beta }_l-\beta _l) \overset{d}{\rightarrow }\ \mathcal {N} (0, \sigma _{\beta _l}^2)$ individually under some mild conditions [46, 48]. We denote the corresponding variance estimators as $\hat{\sigma }_{\beta _l}^2$. Then *p*-values can be computed as follows:

$$\begin{aligned} P_{\hat{\beta }_l}=2\cdot \left( 1- \Phi (|\hat{\beta }_l|/\hat{\sigma }_{\beta _l})\right) , \qquad l\in [q] \end{aligned}$$

where $\Phi (\cdot )$ denotes the cumulative distribution function of the standard normal distribution $\mathcal {N} (0, 1)$.

Second, to estimate each column of the parameter matrix $\varvec{\Theta }$ in the multi-response mediation model defined in Eq. (2), we employ a column-wise regression strategy. For each sub-regression problems, we similarly utilize the *Decorrelating* & *Debiasing* approach after removing the possible observed baseline covariates $\textbf{Z}$ by projection as before. Since the $q$ sub-regression problems share the same predictor $\varvec{X}$ and the calculation of $\textbf{v}_j$ ($j=1,\ldots ,p$) solely depends on $\varvec{X}$, we first compute $\textbf{v}_j$ following Eq. (11). This computation is the most time-consuming aspect of obtaining the *Decorrelating* & *Debiasing* estimator. Fortunately, we only need to compute this step once. Meanwhile, to address the computational challenges posed by large-scale datasets in multi-omics studies, we leverage parallel computing techniques to accelerate the computation for the $q$ sub-regression problems. This approach enables us to efficiently calculate the point and variance estimators for the coefficients $\theta _{kl}$ (where $k \in [p]$ and $l\in [q]$), denoted as $\hat{\theta }_{kl}$ and $\hat{\sigma }_{\theta _{kl}}^2$, respectively. To assess the statistical significance of the coefficient estimates, we calculate the *p*-values using the formula:

$$\begin{aligned} P_{\hat{\theta }_{kl}} =2\cdot \left( 1- \Phi (|\hat{\theta }_{kl}|/\hat{\sigma }_{\theta _{kl}})\right) , \qquad k\in [p],\quad l\in [q]. \end{aligned}$$

Third, we employ the *MinScreen* procedure to screen the total $p \cdot q$ possible causal paths [50]. The screened causal paths by *MinScreen* are defined as the top *K* significant paths: $\hat{\mathcal {S}}_0 {:=} \{(k, l): P_{kl}^{min} \le \alpha _{1}, k \in [p], l \in [q]\}$ where $P_{kl}^{min}=\min \{P_{\hat{\theta }_{kl}},P_{\hat{\beta }_l}\}$ and $\alpha _1$ is chosen such that $|\hat{\mathcal {S}}_0|=K$. This preliminary step eliminates the least promising causal paths before calculating the final *p*-value for $H_{0,kl}$. By doing so, it effectively reduces the computational burden in the subsequent multiple testing phase.

Lastly, we apply the joint significance test (JST), also known as the MaxP test [19], to obtain the *p*-value for the null hypothesis $H_{0,kl}: \theta _{kl} \beta _l = 0$ which tests for no indirect effect, for $(k,l) \in \hat{\mathcal {S}}_0$. The *p*-values for JST are defined as

$$\begin{aligned} P_{kl}^{max}=\max \{P_{\hat{\theta }_{kl}},P_{\hat{\beta }_l}\}, (k,l)\in \hat{\mathcal {S}}_0. \end{aligned}$$

We then sort the JST *p*-values and denote them as $p_{(i)},i=1,\cdots ,K$, the notation for order statistics by convention. To protect the FDR at the nominal level $\alpha$, we find the data-driven *p*-value rejection threshold $P^\star$ using the BH procedure [51]. The threshold $P^\star$ is determined as

$$\begin{aligned} P^{*}=\max \left\{ p_{(i)}:\frac{K\cdot p_{(i)}}{\sum _{j=1}^{K} \mathbb {I}(p_j\le p_{(i)})} \le \alpha , i=1,\cdots , K \right\} . \end{aligned}$$

Finally, we define the set containing statistically significant non-zero path-specific effects as $\hat{\mathcal {S}}=\{(k,l): P_{kl}^{max}\le P^{*}, (k,l)\in \hat{\mathcal {S}}_0\}$. In this article, we evaluate HILAMA and other competitors by FDR and Power, which are defined as follows:

$$\begin{aligned} \text {FDR}=\mathbb {E}\left[ \frac{| \mathcal {\hat{S}} \cap \mathcal {S}^c|}{| \mathcal {\hat{S}}|}\right] , \quad \text {Power}=\mathbb {E}\left[ \frac{|\mathcal {S} \cap \mathcal {\hat{S}} |}{|\mathcal {S}|}\right] , \end{aligned}$$

where $\mathcal {S}=\{(k,l): \theta _{kl}\beta _l\ne 0,k\in [p], l \in [q]\}$ represents the true non-zero effect path-specific set and $\mathcal {S}^c=\{(k,l): \theta _{kl}\beta _l = 0,k\in [p], l\in [q]\}$ represents the zero effect path-specific set.

## Simulation studies

### Simulation design

In this section, we assess if HILAMA is capable of controlling the FDR with sufficient power across a wide range of simulation settings. The performance is compared against various other approaches. As a baseline benchmark, we employ the univariate Baron & Kenny method (abbreviated as BK) [8] for every possible individual exposure-mediator pair, using the R package ${\textbf {mediation}}$. We also consider methods that only allow a *univariate* exposure and high-dimensional mediators, including HIMA [26], HDMA [27] and HIMA2 [28]. To compare the results under a nominal level of 0.1, we made minor modifications to the corresponding R packages **HIMA** and **HDMA**. We analyzed each individual exposure separately and then aggregated the results. Relevant details are provided in the Supplementary Information. Finally, we compare two penalized methods developed for multiple exposures and mediators. Specifically, for the method “mvregmed” [49], we apply the R package **regmed**. While for the method developed by Zhao et al. [42] (abbreviated as ZY) [42], we implement their penalized regression algorithm and omit the dimension reduction step for comparison. Here, we only compare the two penalized methods in **simulation 2** introduced below due to their slow running time.

We first generate the exposure data $\textbf{X}_i (i=1,\cdots ,n)$ according to model (3). The observed baseline covariates $\textbf{Z}_i \in \mathbb {R}^r$, latent confounders $\textbf{H}_i\in \mathbb {R}^{s}$ and the elements of $\varvec{\Phi }_1 \in \mathbb {R}^{r\times p}, \varvec{\Psi }_1 \in \mathbb {R}^{s\times p}$ are independently drawn from the standard normal distribution. The unconfounded components $\textbf{E}_{X,i}$ are drawn from $\mathcal {N}_p (0,\varvec{\Sigma }_E)$, where $\varvec{\Sigma }_{E,kl}=\rho ^{|k-l|} (k,l\in [p])$. The parameter $\rho$ controls the strength of correlation among exposures, and it takes values in the range [0, 1).

Similarly, we generate the the mediator data $\textbf{M}_i (i = 1,\cdots ,n)$ according to model (2). The noise term $\textbf{E}_{M,i}$ are drawn from $\mathcal {N}_q(0,\textbf{I})$ and the confounding effect matrix $\varvec{\Phi }_{2},\varvec{\Psi }_{2}$ are drawn from $\xi \cdot \mathcal {N}(\eta ,1)$, where $\xi$ is a Rademacher random variable, i.e. $P(\xi =1)=P(\xi =-1)=\frac{1}{2}$. Then, for the signal coefficient matrix $\varvec{\Theta }\in \mathbb {R}^{p\times q}$, we randomly choose $p\cdot r_p$ rows having non-zero elements, and choose $\delta$ non-zero elements separately in each of these rows, where $\delta$ follows uniform distribution on $\{5,6,\cdots ,20\}$. The non-zero elements in $\varvec{\Theta }$ follow the distribution $\xi \cdot \mathcal {N}(0.8,0.1)$.

Finally, we generate the outcome data $Y_i (i=1,\cdots ,n)$ according to model (1). The coefficients $\varvec{\gamma }$ are randomly sampled from a distribution $\xi \cdot \mathcal {N}(0.8, 0.1)$, with a total of $p \cdot r_p$ non-zero elements. Similarly, the coefficients $\varvec{\beta }$ are chosen from the same distribution, with $q \cdot r_q$ non-zero elements. To determine the active location in $\varvec{\beta }$, we define $\mathcal {A}^c$ as the set of columns in $\varvec{\Theta }$ with zero elements ($\Vert \Theta _{.l}\Vert _1=0$), and $\mathcal {A}$ as the set of columns in $\varvec{\Theta }$ with non-zero elements ($\Vert \Theta _{.l}\Vert _1\ne 0$). From $\mathcal {A}^c$, we randomly choose $s_{01}$ elements with equal probability, where $s_{01}=\min \{0.2\cdot q\cdot r_{q},|\mathcal {A}^c|\}$. While from $\mathcal {A}$, we randomly choose $s_{11}$ elements with unequal probability, where $s_{11}=q\cdot r_{q} - s_{01}$. The selection probability of $l \in \mathcal {A}$ is determined by $\frac{\Vert \varvec{\Theta }_{.l}\Vert _0}{\Vert \varvec{\Theta }\Vert _0}$, which represents the proportion of non-zero elements in column *l* relative to all non-zero elements in $\varvec{\Theta }$. The confounding effects $\varvec{\phi }$ are drawn from $\xi \cdot \mathcal {N}_s(\eta ,\textbf{I})$, and the noise terms $\epsilon _{Y,i}$ are drawn from $\mathcal {N}(0,1)$.

For all the simulations below, we fix the sparsity proportions as $r_p=r_{q}=0.1$, the dimension of baseline covariates $r=3$, and latent confounders $s = 2$. Additionally, we set $K = 0.1pq$, the nominal FDR level at the $\alpha = 0.1$ and all the simulation results are averaged over 100 Monte Carlo replications.

### Simulation results

**Simulation 1.** In the first simulation, we test the stability of our model under various scenarios. We evaluate the impact of changes in sample size ($n \in \{200,400\}$), exposure dimension ($p \in \{100,200,400\}$), mediator dimension ($q \in \{50,100,200\}$), correlation size among exposures ($\rho \in \{0.4,0.8\}$), and magnitude of latent effects ($\eta \in \{0.5, 1.5\}$).

**Fig. 3:**
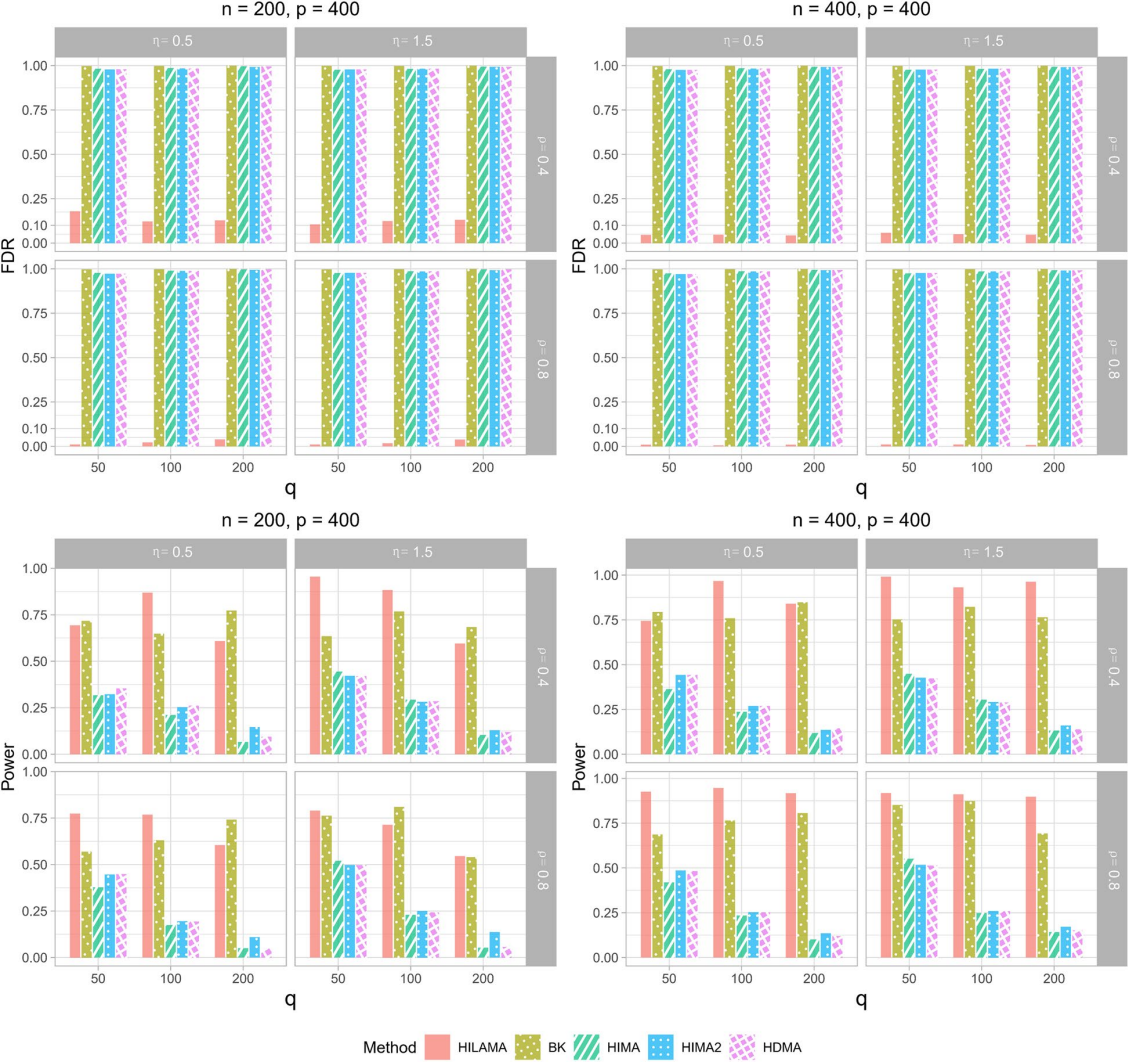
Comparison results of (**A**) (First row) Empirical False Discovery Rate (FDR) and (**B**) (Second row) Empirical Power for different methods in Simulation 1. $\eta$ represents latent confounding effect and $\rho$ represents the correlation size among exposures. Here we only present $p=400$. All results are averaged over 100 replications under the nominal FDR level of 0.1

For simplicity, we only present scenarios for $p = 400$ in Fig. 3. For the total 72 different settings, we present the average value of empirical FDR and Power in Supplementary Information. From Fig. 3A, only HILAMA controls the FDR at the nominal level $\alpha = 0.1$ in all scenarios, whereas the other three methods all fail to do so. The reasons for their lack of control are due to their failure to correct for the effect of latent confounding, and their inability to accommodate high-dimensional exposure and mediator settings. Turning to the power, by reading Fig. 3B, we can easily see that HILAMA achieves the highest power in larger sample sizes ($n=400$). However, in smaller sample sizes ($n=200$), the statistical power decreases as correlation coefficient $\rho$ increases. The impact of the aforementioned parameters on the power of HILAMA is generally diminished in larger sample sizes. The powers of the other three methods, on the other hand, are essentially meaningless since their FDRs are all close to 1. Moreover, the point estimates of mediation effects produced by HILAMA exhibit substantially less bias compared to other methods, as measured by $\frac{\sum _{(k,l)\in \mathcal {S}}|\theta _{kl}\beta _l-\hat{\theta }_{kl}\hat{\beta }_l|}{|\mathcal {S}|}$ (see Supplementary Information).

#### Remark 2

As noted by one reviewer, it may be more appropriate to consider scenarios in which methods like HIMA2 perform adequately and to demonstrate how HILAMA performs in that context. To provide a more comprehensive comparison of the advantages and disadvantages of each approach, we considered scenarios including single exposure and those without hidden confounders in the Supplementary Information. In particular, we observe that methods such as HIMA2 can effectively control the FDR and achieve high power only in the absence of hidden confounding; however, they struggle with correlated exposures or hidden confounders. In contrast, HILAMA underperforms in controlling the FDR and exhibits lower power compared to other methods when dealing with a limited number of exposures.

**Simulation 2.** In the second simulation, we evaluate the impact of latent confounding density on the performance of HILAMA and compare it with the two penalized methods developed for multivariate exposures and mediators, as mentioned earlier. The denseness of latent confounding is measured as the proportion $(1 - r_h)$ of zero entries in each row of matrices $\varvec{\Psi }_1$ and $\varvec{\Psi }_2$. If $r_h = 0$, then $\varvec{\Psi }_1 = \varvec{\Psi }_2 = 0$, amounting to no latent confounding; whereas if $r_h = 1$, all exposures and mediators are confounded by latent confounders, as depicted in simulation 1. Here, we vary only $r_h \in \{0, 0.1, 0.2, \cdots , 0.9, 1\}$ while holding $n =300, p = q = 100, \rho = 0.6$ and $\eta = 1$.

To compare our *p*-value based method with the penalized methods mvregmed and ZY, here we assume that the actual number of active pairs is known. We select the top $|\hat{\mathcal {S}}|$ pairs that control the FDR at the level 0.1 and compare their power. If the FDR cannot be controlled at the 0.1 level, we choose the cut-off point associated with the lowest FDR and calculate the corresponding power.

**Fig. 4:**
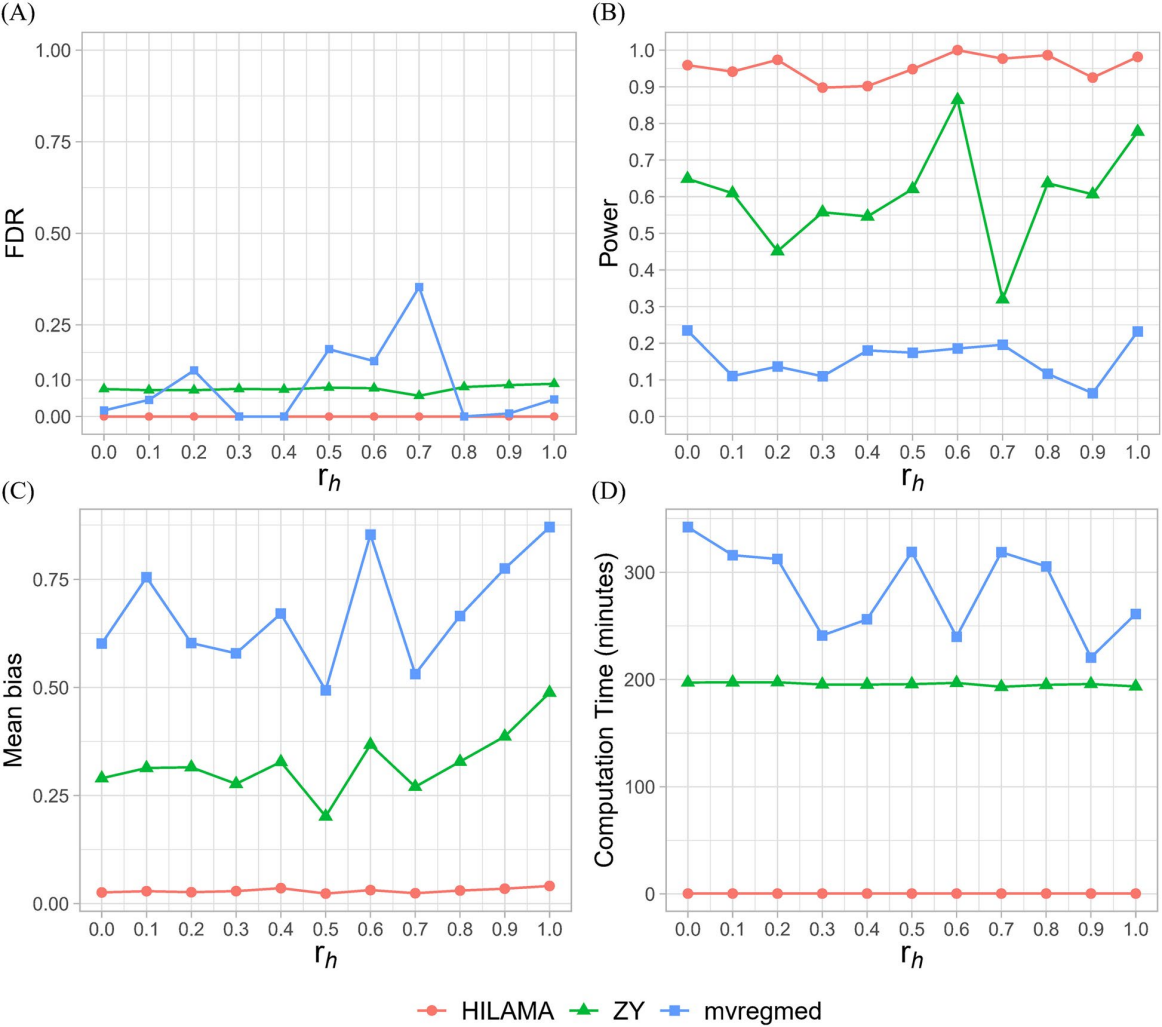
Comparison results of different methods across varied confounding density $r_h$. **A** Empirical FDR, **B** Empirical Power, **C** Mean bias and **D** Computation time (minutes). All results are averaged over 100 replications. Fix $n=300,p= q =100,\rho =0.6,\eta =1,r_p=r_{pq}=0.1,r=3$ and $s=2$

Figure 4A indicates that both HILAMA and ZY can manage the FDR at the 0.1 level even when some observed variables are confounded by latent confounders. Additionally, HILAMA exhibits the highest power compared to the other two methods across all confounding density setting, as shown in Fig. 4B. However, mvregmed does not effectively control the FDR in certain situations, and its power is relatively low. Furthermore, Fig. 4C demonstrates that HILAMA again has the minimum mean bias compared to the other two competing methods, mvregmed and ZY. Specifically, Fig. 4D shows that although the ZY method achieves good FDR control and power performance, it takes hundreds of times longer to compute than HILAMA, even in this low-dimensional setting.

**Simulation 3.** In the third simulation, our aim is to further investigate the impact of signal strength and hidden confounding density on HILAMA. Specifically, we examine the distribution of $\varvec{\Theta }$, $\varvec{\beta }$, and $\varvec{\gamma }$ by allowing their non-zero components to follow $\xi \cdot \mathcal {N}(\kappa ,0.1)$, where $\kappa \in \{0.1, 0.15, \cdots , 1.45, 1.5\}$. Additionally, we consider different values for the confounding density $r_h \in \{0, 0.1, 0.2, 0.8, 1\}$ and the exposure dimension $p \in \{100,300\}$, while maintaining $n=300,q=100,\rho =0.6$, and $\eta =1$.

As depicted by Fig. S3 in Supplementary Information, when the confounding density is small ($r_h=0.1,0.2$), HILAMA fails to effectively control the FDR. Conversely, when the confounding density increases ($r_h=0.8,1$), it successfully controls the FDR across all signal strength levels. Notably, even in the absence of hidden confounding, HILAMA exhibits good FDR control. Moreover, as shown in the second column of Fig. S3, the power of HILAMA increases with the signal strength, approaching 1 for all levels of confounding density.

## Data application

In this section, we apply HILAMA to a real multi-omics dataset collected by the ADNI. Before delving into the details, we emphasize that this data analysis should be viewed as at most exploratory rather than confirmatory nature. It is highly likely that the linearity assumption imposed in the Structural Equation Model may not be a good approximation of the reality.

Alzheimer’s disease (AD) is an irreversible and complex neurological disease that affects millions of individuals worldwide. Currently, approximately 6.7 million Americans aged 65 years and older live with AD, and this number is projected to dramatically increase to 13.8 million by the year 2060 [54]. AD is characterized by progressive memory loss and other cognitive impairments resulting from the accumulation of amyloid-$\beta$ (A$\beta$) and tau proteins in the brain, leading to neurodegenerative symptoms [55]. Specifically, the model of AD pathophysiology outlines a chronological sequence of events in which the formation of A$\beta$ plaques is followed by the deposition of abnormal tau aggregates, subsequent neuronal dysfunction and neurodegeneration, including structural atrophy of cerebral regions such as the hippocampus. Ultimately, this sequence results in cognitive impairment and dementia [56–58]. This model of the temporal sequence of events has been continuously supported by new evidence [59–62].

Unfortunately, there is currently no effective treatment for AD, underscoring the significance of early diagnosis and comprehending the disease’s pathogenesis. Therefore, it is crucial to develop effective interventions to prevent, slow down, or even cure this disease through biomedical research. With this in mind, the Alzheimer’s Disease Neuroimaging Initiative (ADNI, adni.loni.usc.edu) was established in 2003. Its primary goals are to develop biomarkers for AD, enhance the understanding of its pathophysiology, and improve early detection using various modalities such as magnetic resonance imaging (MRI), positron emission tomography (PET), functional magnetic resonance imaging (fMRI), as well as clinical and neuropsychological assessments.

Here, we utilize the HILAMA approach to examine the connection between proteins in the cerebrospinal fluid (CSF), whole-brain regions, and cognitive behavior. Our aim is to identify critical biological pathways associated with AD by utilizing data from the ADNI database. The CSF proteomics data is acquired using a highly specific and sensitive technique called targeted liquid chromatography multiple reaction monitoring mass spectrometry (LC/MS-MRM), resulting a list of 142 annotated proteins derived from 320 peptides. Additionally, the brain imaging data is obtained through anatomical magnetic resonance imaging (MRI), and volumetric measurements are extracted from 145 brain regions-of-interest (ROI) [63]. To assess the relationship between the aforementioned variables and cognitive function, we consider the composite memory score as the response. This score is measured using the ADNI neuropsychological battery, with higher scores indicating better cognitive function. In our model, we treat the 142 proteins as exposures ($\textbf{X}$), the 145 brain regions as mediators ($\textbf{M}$), and the memory score as the outcome ($\textbf{Y}$). For this study, we focus on a total of 287 subjects who have both proteomics and imaging data available. These subjects consist of 86 cognitively normal individuals (CN), 135 patients with mild cognitive impairment (MCI), and 66 AD patients. To account for potential confounding effects, we include covariates such as age, years of education. For more detailed information on these baseline covariates, please refer to Table 1.

**Table 1:** Frequencies and descriptive statistics for demographic and clinical variables in the sample

Disease status	CN	MCI	AD	Total
Number	86	135	66	287
Memory score	0.76 ± 0.39	−0.16 ± 0.43	−0.79 ± 0.35	−0.02 ± 0.7
Age	75.9 ± 5.54	74.8 ± 7.35	75.1 ± 7.57	75.22 ± 6.9
Years of education	15.6 ± 3.0	16 ± 2.96	15.1 ± 2.96	15.69 ± 2.97

Values except in the second line are expressed as mean ± standard deviation

*CN* Cognitively Normal individuals, *MCI* Mild Cognitive Impairment individuals, *AD* Alzheimer’s Disease individuals

Prior to conducting the mediation analysis, we impute some volumetric measures recorded as zero with the corresponding median value in the observed data, and then apply a log-transformation to make the corresponding distribution closer to normal. Subsequently, we standardize the baseline covariates data, protein data and MRI data to have a mean of zero and a standard deviation of one, while only centering the outcome cognitive score to have a mean of zero. In Supplementary Information, we visualize the singular values of the protein and MRI data, allowing us to assess the potential presence of latent confounders in the outcome model and mediator model. By examining Fig. S4., we observe the presence of three and two significantly larger singular values. This finding suggests a distinct spiked structure, indicating the possible presence of latent confounders as depicted in the model (2) and (3).

**Fig. 5:**
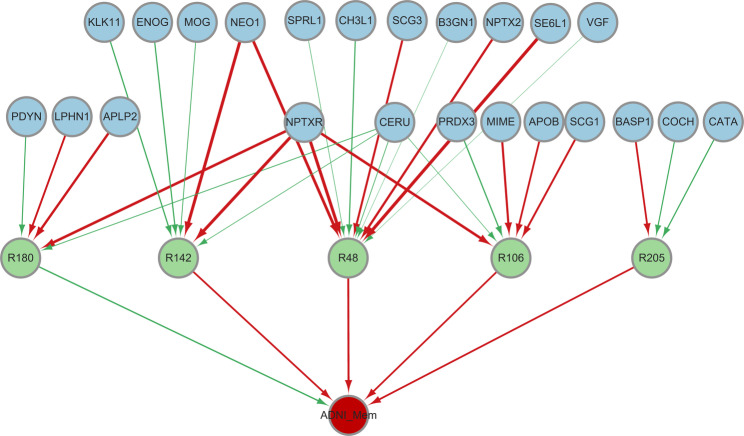
The significant causal paths using proteomics-radiomics data. Blue nodes represent the proteins as exposures, green nodes represent the brain regions as mediators and the red node represents the memory score as the outcome. Red lines indicate positive estimates while green lines represent negative estimates. Line thickness corresponds to the effect size. Blue nodes are arranged in two rows for visual clarity and positioning carries no extra information

Following the preprocessing of data, we apply our method to the processed data. However, after implementing the BH procedure, no significant paths are obtained when controlling the FDR at a nominal level of 0.1. In order to obtain meaningful results, we relax the criterion and set the significance threshold for *p*-values to 0.05 without applying multiple correction. Consequently, we identify 30 significant causal paths, corresponding to 23 proteins and 5 brain regions. In Fig. 5, we visualize the significant causal paths. The estimated path effects, including the $\theta _{kl}$ and $\beta _l$, are presented in the Supplementary Information.

Our study has identified several brain regions associated with cognitive impairment and AD. Among them, R48 (left hippocampus) plays a crucial role in learning and memory, and is particularly vulnerable to early-stage damage in AD [64]. Importantly, hippocampal atrophy has been universally recognized and validated as the most reliable biomarker for AD [65]. Another crucial region in cognition is R106 (right angular gyrus), which is associated with language, spatial, and memory functions [66, 67]. The aging process leads to structural atrophy in the angular gyrus, which is linked to subjective and mild cognitive impairments, as well as dementia [68, 69]. Additionally, another significant region R142 (right middle frontal gyrus), exhibits a positive correlation between enhanced connectivity within this area and the cognitive decline observed in individuals with mild AD symptoms [70]. Furthermore, heightened connectivity within the middle frontal gyrus may alleviate the consequences of reduced connectivity in other regions of the cognitive control network among AD patients [71]. Furthermore, R205 (left triangular part of the inferior frontal gyrus) and R180 (right planum polare) are also associated with AD and cognitive impairment. However, further investigation is necessary to comprehensively elucidate the roles of these regions in AD pathology and cognitive function.

Several proteins have been identified as potentially critical biomarkers for AD. NPTX2 (Neuronal pentraxin-2) and NPTXR (Neuronal pentraxin receptor) are proteins that bind to glutamate receptors, contributing to synaptic plasticity. Reductions in NPTX2 have been linked to disruptions of the pyramidal neuron-PV interneuron circuit in an AD mouse model [72]. SE6L1 (Seizure 6-like protein) is a potential neuronal substrate of the AD protease BACE1, which is a major drug target in AD [73]. Aberrant function of SE6L1 may lead to movement disorders and neuropsychiatric diseases [74]. Overexpression of the neuropeptide precursor VGF has been found to partially rescue A$\beta$ mediated memory impairment and neuropathology in a mouse model, indicating a protective function against the development and progression of AD [75]. CERU (Ceruloplasmin), a ferrous oxidase enzyme, plays an important role in regulating iron metabolism and redox reactions. Experiments using AD mouse models have shown that ceruloplasmin depletion exacerbates memory impairment, promotes iron accumulation, and restoration of its expression alleviates A$\beta$-induced neuronal damage in the hippocampus [76]. CH3L1 (Chitinase-3-like protein 1) is a biomarker for its ability to detect neuroinflammation and diagnose AD. Elevated levels of CHI3L1 in the CSF can be detected in the early stages of AD, even before the onset of cognitive symptoms [77]. SCG3 (Secretogranin-3), is a member of the granin family involved in neurotransmitter storage and secretion. In vitro studies have highlighted the critical involvement of SCG3 in neuroendocrine regulation, neuronal communication, and neurotransmitter release [78]. NEO1 (Neogenin) is a transmembrane receptor involved in adult neurogenesis. Experimental studies have demonstrated the essential role of neogenin in promoting neurogenesis in the adult hippocampus and preventing depressive-like behavior [79]. APOB (Apolipoprotein B-100) is a recognized risk factor for AD that potentially impact both brain aging and cognitive function. Experimental studies using APOB-100 transgenic mice models have demonstrated that excessive expression of APOB results in memory decline [80]. Moreover, a Mendelian randomization analysis has provide initial evidence suggesting that APOB contributes to an increased risk of developing Alzheimer’s disease [81]. CATA (Catalase) plays a significant role in the intracellular interactions between catalase and amyloid in A$\beta$-induced oxidative stress. This interaction leads to the accumulation of hydrogen peroxide and the onset of oxidative stress conditions in the hippocampus, thereby contributing to the pathogenesis of Alzheimer’s disease [82]. PRDX3 functions as a crucial mitochondrial antioxidant defense enzyme, and its overexpression provides protection against cognitive impairment while reducing the accumulation of A$\beta$ in transgenic mice [83]. Furthermore, its overexpression reduces mitochondrial oxidative stress, attenuates memory impairment induced by hydrogen peroxide and improves cognitive ability in transgenic mice [84]. Recent research has also highlighted the important roles of PRDX3 in neurite outgrowth and the development of AD [85].

As suggested by one reviewer, interaction terms between exposures and mediators are common in mediation analysis. To investigate the possibility of these interaction terms, we applied the recently developed model XMInt [86], which focuses on a single exposure and high-dimensional mediators by employing a sequential regularization-based forward selection approach. We selected one protein at a time as the exposure while utilizing the 145 brain regions as mediators. In our analysis, two proteins were identified across four pathways: NPTXR and CERU. We conducted separate analyses for these two proteins to identify potential interaction terms using the XMInt package with the default parameter setting. When NPTXR was used as the exposure, we identified two brain regions, R123 (Left Fusiform Gyrus) and R144 (Right Middle Occipital Gyrus), as mediators, both of which exhibited interactions with the exposure. In contrast, when CERU was used as the exposure, no mediators or interaction terms were identified.

In summary, our study identified several critical brain regions, such as R48, R106 and R142, that are associated with learning, memory, and recognition. Moreover, we have identified several potential biomarkers for AD, such as NPTX2, NPTXR, SE6L1, CERU, VGF, CH3L1, NEO1, SCG3, PRDX3 etc., most of which are not selected by the method ZY [42]. Nonetheless, it is crucial to note that these findings are only suggestive and further experimental validation is warranted to fully understand their contributions to AD pathology and cognitive function.

## Discussion

In this paper, we propose HILAMA, a new method for high-dimensional mediation analysis, an important statistical task in the analysis of multi-omics datasets increasingly available in biomedical sciences. HILAMA effectively unravels the causal pathway between high-dimensional exposures and a continuous outcome, in the presence of possibly latent/unmeasured confounders. We validate the practical performance of HILAMA through extensive simulations and by applying it to a real ADNI dataset, which allows for the identification of potential biomarkers for Alzheimer’s disease.

HILAMA features several key advantages over previous methods, designed towards better fitting into real-world multi-omics datasets. First, it is the first method to consider both high-dimensional exposures and high-dimensional mediators in the presence of latent confounders without transforming exposures/mediators into principal components, rendering the analysis results more interpretable. Second, it incorporates a new *Decorrelating* & *Debiasing* method [48] to handle latent/unmeasured confounding and improve coefficient estimation, leading to better FDR control. Third, it employs a *MinScreen* screening procedure [50] to reduce the number of hypotheses being tested, thereby enhancing the statistical power of the tests. Finally, the method is computationally efficient and has implemented parallel computing techniques to handle the ever-increasing size and dimension of modern multi-omics datasets.

To conclude, we point out several venues for future research. First, HILAMA assumes linear models, which is standard practice in multi-omics studies. However, it will be interesting to generalize it to nonlinear/nonparametric models via nonlinear factor analysis [87], autoencoders [88], kernel methods or deep neural networks [89]. Second, HILAMA assumes that the effects of latent/unmeasured confounders on observables are dense. It may be possible to relax this assumption by extending the randomized data-augmentation scheme for total effect to the mediation analysis setting [34]. Third, interaction terms between exposures and mediators are common in mediation analysis, particularly considering multiple exposures and mediators. Incorporating these interaction terms could provide a more nuanced understanding of the relationships involved and enhance the ability of the model to capture certain nonlinear phenomenon. Finally, other methods of dealing with latent confounding can also be incorporated into HILAMA in its future version, such as the approaches [90] that directly leverage the majority rule [91] or the plurality rule [92, 93]. Overall, these future research directions have the potential to expand the capabilities of HILAMA, allowing for more accurate and robust causal inference in multi-omics studies.

## Conclusion

The proposed HILAMA method integrates the *Decorrelating* & *Debiasing* method and the *MinScreen* screening procedure, thereby further highlighting its superiority over existing methods, particularly in terms of FDR control and statistical power. It introduces a novel approach for elucidating the causal pathway between high-dimensional exposures and a continuous outcome in the presence of latent/unmeasured confounders, thereby enhancing interpretability, a critical aspect in biomedical research.

## Supplementary Information

Supplementary Material 1. Derivations of direct and indirect effects under Latent Structural Equation Modeling. The empirical FDR, power and mean bias for different methods across 72 scenarios in simulation 1 are shown in Fig. S1. Comparison results of empirical FDR and power of different methods under scenarios including single-exposure and those without hidden confounders are shown in Fig. S2. Empirical FDR and power of HILAMA with varied signal strength $\rho$, confounding density $r_h$ and exposure dimension *p* in simulation 3 are shown in Fig. S3. Singular values of standardized exposure and mediator data after projection are shown in Fig. S4. The summary statistics of the significant paths are shown in Table S1.

## Data Availability

An R package implementing our new method HILAMA is publicly available at https://github.com/Cinbo-Wang/HILAMA. Instructions for generating our simulated data can be found at https://github.com/Cinbo-Wang/Simu_HILAMA, which includes the main R-scripts used to generate the simulation data. The ADNI data were obtained from the Alzheimer’s Disease Neuroimaging Initiative (ADNI) database (adni.loni.usc.edu) after applying for access and obtaining approval from the ADNI DPC. For more details, see https://ida.loni.usc.edu/explore/jsp/support/support.jsp.

## Abbreviations

ADNI: Alzheimer’s Disease Neuroimaging Initiative
JST: Joint Significance Test
FDR: False Discovery Rate
LSEM: Latent Structural Equation Modeling
HILAMA: HIgh-dimensional LAtent-confounding Mediation Analysis
BH: Benjamini-Hochberg
BK: Baron & Kenny method
HIMA: High-dimensional Mediation Analysis
HDMA: High-dimensional De-biased Mediation Analysis
SIS: Sure Independence Screening
CSF: Cerebrospinal Fluid
CN: Cognitively Normal individuals
MCI: Mild Cognitive Impairment
AD: Alzheimer’s Disease
