# Supplementary material for: HILAMA: High-dimensional multi-omics mediation analysis with latent confounding
Source: BMC Med Res Methodol. 2025 Oct 24;25:239. doi: 10.1186/s12874-025-02686-z (PMC12551175; doi:10.1186/s12874-025-02686-z)
Supplement: Supplementary file 1 — Supplementary Material 1. Derivations of direct and indirect effects under Latent Structural Equation Modeling. The empirical FDR, power and mean bias for different methods across 72 scenarios in simulation 1 are shown in Fig. S1. Comparison results of empirical FDR and power of different methods under scenarios including single-exposure and those without hidden confounders are shown in Fig. S2. Empirical FDR and power of HILAMA with varied signal strength $\rho$, confounding density $r_h$ and exposure dimension p in simulation 3 are shown in Fig. S3. Singular values of standardized exposure and mediator data after projection are shown in Fig. S4. The summary statistics of the significant paths are shown in Table S1. [file 12874_2025_2686_MOESM1_ESM.pdf]

Supplementary Materials

Derivations of direct and indirect effects under LSEM (1) and (2)

We follow the potential outcomes framework [1, 2] to decompose the effect of exposures on the outcome. Specifically, we denote $Y(\mathbf{X}, \mathbf{M})$ as the potential outcome when the exposure $\mathbf{X}$ is set to $\mathbf{X} = (x_1, \cdots, x_p)'$, the mediator $\mathbf{M}$ is set to $\mathbf{M} = (m_1, \cdots, m_q)'$. Formally, we define $\mathbf{X}_{-k} = (x_1, \cdots, x_{k-1}, x_{k+1}, \cdots, x_p)'$ and use $M_l(x_k, \mathbf{X}^*_{-k})$ as the potential value of the l-th mediator where the k-th exposure is set to x_k and the other exposures are set to $\mathbf{X}^*_{-k}$. We denote the baseline adjusted covariates as $\boldsymbol{Z}_0 = (\boldsymbol{Z}, \mathbf{H})$, where $\boldsymbol{Z}$ represents the observed confounders, $\mathbf{H}$ represents the potential latent confounders, and $\mathbf{X}^*$ as the reference level of exposure. Then, the average total effect (TE) of the k-th exposure on the outcome is defined as $\mathbb{E}[Y(x_k, \mathbf{X}^*_{-k}, \mathbf{M}(x_k, \mathbf{X}^*_{-k}))|\boldsymbol{Z}_0] - \mathbb{E}[Y(x^*_k, \mathbf{X}^*_{-k}, \mathbf{M}(x^*_k, \mathbf{X}^*_{-k}))|\boldsymbol{Z}_0]$; the average natural direct effect (NDE) of X_k on Y is $\mathbb{E}[Y(x_k, \mathbf{X}^*_{-k}, \mathbf{M}(\mathbf{X}^*))|\boldsymbol{Z}_0] - \mathbb{E}[Y(\mathbf{X}^*, \mathbf{M}(\mathbf{X}^*))|\boldsymbol{Z}_0]$; the average natural indirect effect (NIE) of X_k on Y is $\mathbb{E}[Y(x_k, \mathbf{X}^*_{-k}, \mathbf{M}(x_k, \mathbf{X}^*_{-k}))|\boldsymbol{Z}_0] - \mathbb{E}[Y(x_k, \mathbf{X}^*_{-k}, \mathbf{M}(\mathbf{X}^*))|\boldsymbol{Z}_0]$. Among the three effects, we have the following relationships:

$$
\begin{aligned}
&\underbrace{\mathbb{E}[Y(x_k, \mathbf{X}^*_{-k}, \mathbf{M}(x_k, \mathbf{X}^*_{-k}))|\boldsymbol{Z}_0] - \mathbb{E}[Y(x^*_k, \mathbf{X}^*_{-k}, \mathbf{M}(x^*_k, \mathbf{X}^*_{-k}))|\boldsymbol{Z}_0]}_{\mathrm{TE}_k} \\
&= \underbrace{\mathbb{E}[Y(x_k, \mathbf{X}^*_{-k}, \mathbf{M}(\mathbf{X}^*))|\boldsymbol{Z}_0] - \mathbb{E}[Y(\mathbf{X}^*, \mathbf{M}(\mathbf{X}^*))|\boldsymbol{Z}_0]}_{\mathrm{NDE}_k} \\
&+ \underbrace{\mathbb{E}[Y(x_k, \mathbf{X}^*_{-k}, \mathbf{M}(x_k, \mathbf{X}^*_{-k}))|\boldsymbol{Z}_0] - \mathbb{E}[Y(x_k, \mathbf{X}^*_{-k}, \mathbf{M}(\mathbf{X}^*))|\boldsymbol{Z}_0]}_{\mathrm{NIE}_k}.
\end{aligned}
$$

To identify the above NDE and NIE, we need the following standard ignorability assumption [3]:

(C1) $Y(\mathbf{X}, \mathbf{M}) \perp X_k|\boldsymbol{Z}_0$ for $\forall \mathbf{X}, \mathbf{M}, k \in [p]$, i.e. no unmeasured confounding between the exposures and the outcome;

(C2) $Y(\mathbf{X}, \mathbf{M}) \perp M_l|\boldsymbol{Z}_0$ for $\forall \mathbf{X}, \mathbf{M}, l \in [q]$, i.e. no unmeasured confounding between the mediators and the outcome;

(C3) $M_l(\mathbf{X}) \perp X_k|\boldsymbol{Z}_0$ for $\forall \mathbf{X}, k \in [p], l \in [q]$, i.e. no unmeasured confounding between the exposures and the outcome;

(C4) $Y(\mathbf{X}, \mathbf{M}) \perp M_l(\mathbf{X}^*)|\boldsymbol{Z}_0$ for $\forall \mathbf{X}, \mathbf{X}^*, \mathbf{M}, l \in [q]$, i.e. no unmeasured confounding between the mediators and the outcome that is itself affected by the exposures.

From LSEM (1) and (2), we can express the potential mediator $M_l(x_k, x^*_{-k})$ and potential outcome $Y(x_k, \mathbf{X}^*_{-k}, \mathbf{M}(x_k, \mathbf{X}^*_{-k}))$ as follows:

$$M_l(x_k, \mathbf{X}^*_{-k}) = x_k\theta_{kl} + \mathbf{X}^{*T}_{-k}\boldsymbol{\theta}_{-k,l} + \boldsymbol{Z}^\top\boldsymbol{\Phi}_{2,\cdot,l} + \boldsymbol{H}^\top\boldsymbol{\Psi}_{2,\cdot,l} + E_{M,l}, \quad l \in [q] \tag{1}$$

$$Y(x_k, \mathbf{X}^*_{-k}, \mathbf{M}(x_k, \mathbf{X}^*_{-k})) = x_k\gamma_k + \mathbf{X}^{*T}_{-k}\boldsymbol{\gamma}_{-k} + \sum_{l=1}^{q} M_l(x_k, \mathbf{X}^*_{-k})\beta_l + \boldsymbol{Z}^\top\boldsymbol{\phi} + \boldsymbol{H}^\top\boldsymbol{\psi} + \epsilon \tag{2}$$

Then, the (average) natural direct effect of exposure X_k on outcome when the value of that exposure is manipulated from x_k to x^*_k, denoted by $\mathrm{NDE}_k(x_k, x^*_k)$, can be derived directly based on equation (2):

$$
\begin{aligned}
\mathrm{NDE}_k(x_k, x^*_k) &= \mathbb{E}[Y(x_k, \mathbf{X}^*_{-k}, \mathbf{M}(\mathbf{X}^*))|\boldsymbol{Z}_0] - \mathbb{E}[Y(\mathbf{X}^*, \mathbf{M}(\mathbf{X}^*))|\boldsymbol{Z}_0] \\
&= \gamma_k(x_k - x^*_k), \quad k \in [p].
\end{aligned}
$$

Similarly, the natural indirect effect of exposure X_k on outcome, denoted by $\text{NIE}_k(x_k, x_k^*)$, can be derived as follows:

$$\begin{aligned}\text{NIE}_k(x_k, x_k^*) &= \mathbb{E}[Y(x_k, \mathbf{X}^*_{-k}, \mathbf{M}(x_k, \mathbf{X}^*_{-k}))|\boldsymbol{Z}_0] - \mathbb{E}[Y(x_k, \mathbf{X}^*_{-k}, \mathbf{M}(\mathbf{X}^*))|\boldsymbol{Z}_0] \\ &= \sum_{l=1}^{q} \mathbb{E}[M_l(x_k, \mathbf{X}^*_{-k}) - M_l(x_k^*, \mathbf{X}^*_{-k})|\boldsymbol{Z}_0]\beta_l \\ &= \sum_{l=1}^{q} (x_k - x_k^*)\theta_{kl}\beta_l.\end{aligned}$$

Supplementary tables and figures

Simulation

Simulation 1: In the main text, we compare HIMA, HIMA2, and HDMA at a nominal False Discover Rate (FDR) level of 0.1. Several modifications are detailed below, along with the methods for calculating FDR and power:

- HIMA defaults to returning the mediators selected through penalized regression, along with their corresponding p-values for the indirect effect adjusted using the Benjamini-Hochberg (BH) method. To ensure a fair comparison, unadjusted p-values of the selected mediators are provided. The variable selection process can be viewed as a method of screening similar with *MinScreen* in the main text. When aggregating the p-values obtained from p iterations during multiple testing, we apply the BH correction to calculate the FDR and power.
- HDMA defaults to returning the p-values $P_{kl}^{\max} = \max\{P_{\beta_l}, P_{\theta_{kl}}\}$ for the mediators corresponding to $P_{\beta_l} < 0.05$. To ensure a fair comparison, mediators corresponding to $P_{\beta_l} < 0.1$ are returned. Similarly, this is viewed as a screening step, and the BH correction is applied to the combined p-values before calculating the FDR and power.
- HIMA2 defaults to returning the uncorrected p-values for mediators identified at a significance level of 0.05 after multiple corrections. To ensure a fair comparison, we adjust the significance level to 0.1 for the identified mediators. After combining the p-values obtained from p iterations, the BH correction is applied to the merged p-values before calculating the FDR and power.

Results from simulation 1 are shown below:

(a)

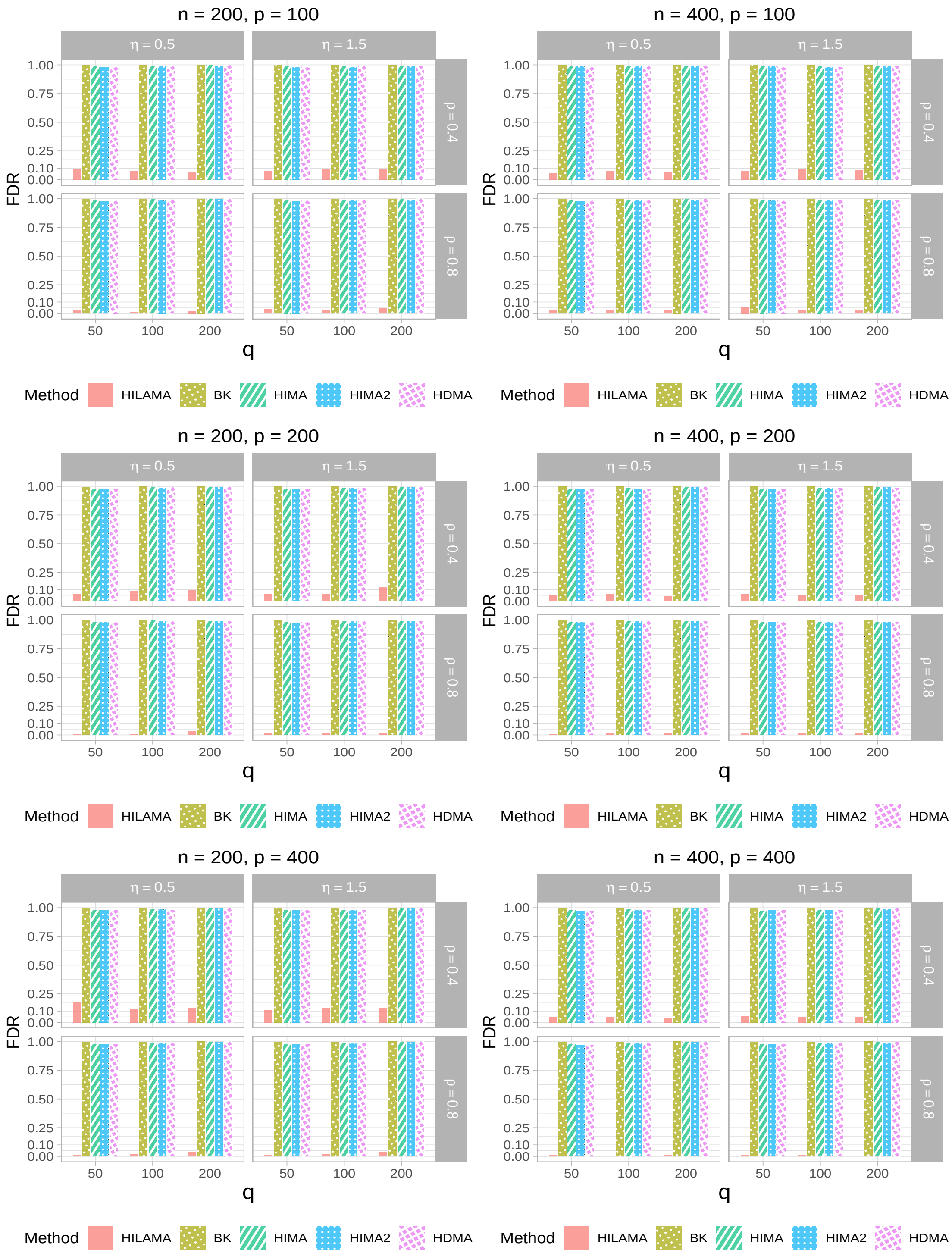

Fig. S1. Comparison results of (a) Empirical False Discovery Rate (FDR), (b) Empirical Power and (c) Empirical Mean bias for different methods in **simulation 1** across 72 scenarios. η represents latent effect and ρ represents the correlation size among exposure. All the results are averaged over 100 replications under the nominal FDR level of 0.1.

(b)

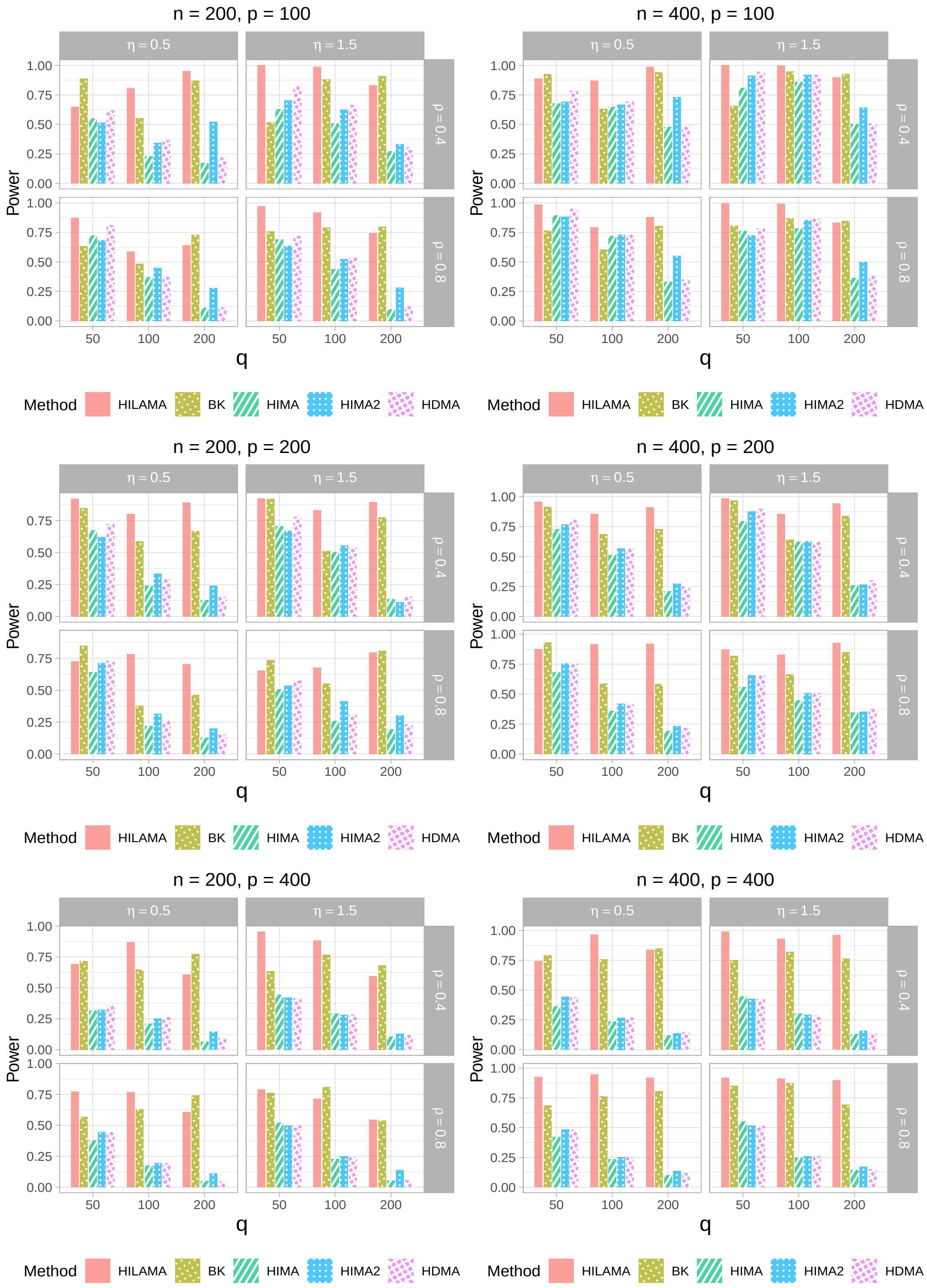

Fig. S1. Comparison results of (a) Empirical False Discovery Rate (FDR), (b) Empirical Power and (c) Empirical Mean bias for different methods in **simulation 1** across 72 scenarios. (continued).

(c)

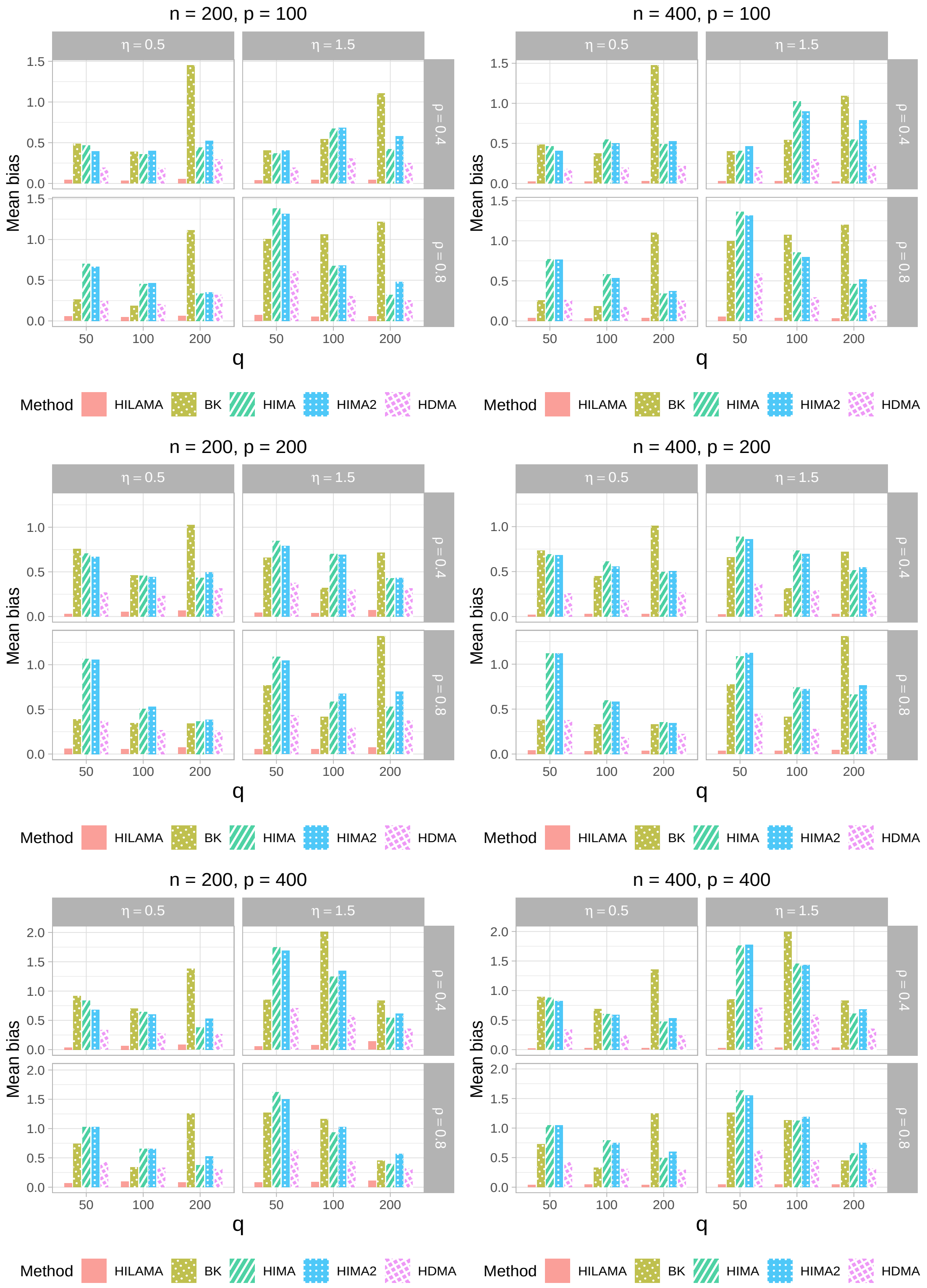

Fig. S1. Comparison results of (a) Empirical False Discovery Rate (FDR), (b) Empirical Power and (c) Empirical Mean bias for different methods in **simulation 1** across 72 scenarios. (continued).

Additional simulation: As noted by one reviewer, it might be more appropriate to consider a scenario in which methods like HIMA2 perform adequately well and to demonstrate how HILAMA compares in that text. To provide a more comprehensive comparison of the advantages and disadvantages of each approach, we considered scenarios including single-exposure and those without hidden confounders. Details are presented below.

Our mediation model is formulated as follows

$$Y_i = \mathbf{X}_i^\top \boldsymbol{\gamma} + \mathbf{M}_i^\top \boldsymbol{\beta} + \mathbf{Z}_i^\top \boldsymbol{\phi} + \mathbf{H}_i^\top \boldsymbol{\psi} + \epsilon_i, \tag{3}$$

$$\mathbf{M}_i = \boldsymbol{\Theta}^\top \mathbf{X}_i + \boldsymbol{\Phi}_2^\top \mathbf{Z}_i + \boldsymbol{\Psi}_2^\top \mathbf{H}_i + \mathbf{E}_{M,i}, \tag{4}$$

$$\mathbf{X}_i = \boldsymbol{\Phi}_1^\top \mathbf{Z}_i + \boldsymbol{\Psi}_1^\top \mathbf{H}_i + \mathbf{E}_{X,i}, \tag{5}$$

where $Y_i \in \mathbb{R}, \mathbf{X}_i \in \mathbb{R}^p, \mathbf{M}_i \in \mathbb{R}^q, \mathbf{Z}_i \in \mathbb{R}^r, \mathbf{H}_i \in \mathbb{R}^s$.

We begin by generating the coefficients as follows

- Each element of $\boldsymbol{\phi}, \boldsymbol{\Phi}_1, \boldsymbol{\Phi}_2$ is drawn from $\xi \cdot \mathcal{N}(0.5, 0.1)$, with $\mathbb{P}(\xi = 1) = \mathbb{P}(\xi = -1) = \frac{1}{2}$.
- Each element of $\boldsymbol{\psi}, \boldsymbol{\Psi}_1, \boldsymbol{\Psi}_2$ is drawn from $Uniform\{[-\eta, \eta]\}$.
- Each non-zero element of $\boldsymbol{\Theta}, \boldsymbol{\gamma}, \boldsymbol{\beta}$ is drawn from $\xi \cdot \mathcal{N}(0.8, 0.1)$, with the selection of its location following the same procedure as described in the main text.

Here, we select $\lceil 0.2p \rceil$ rows of $\boldsymbol{\Theta}$ with non-zero elements, $\lceil 0.1p \rceil$ non-zero elements for $\boldsymbol{\gamma}$, and $\lceil 0.1q \rceil$ non-zero elements for $\boldsymbol{\beta}$. The elements of $\mathbf{Z}_i, \mathbf{H}_i, \mathbf{E}_{M,i}, \epsilon_i$ are drawn independently from $\mathcal{N}(0,1)$, while $\mathbf{E}_{X,i}$ is drawn from $\mathcal{N}_p(0, \boldsymbol{\Sigma})$ with $\Sigma_{i,j} = \rho^{|i-j|}$. Then, $\mathbf{X}_i, \mathbf{M}_i, Y_i$ are generated according to equations (3) - (5). We fix parameters at $q = 200, s = 2, r = 2, n = 400$, varying $p \in \{1, 3, 7, 50, 75, 100\}, \eta \in \{0, 0.2, 0.4, 0.6, \ldots, 1\}$, and $\rho \in \{0, 0.15, 0.3\}$. For HILAMA, we set $K = 0.1pq$ with a nominal FDR of $\alpha = 0.1$. All simulation results are averaged over 100 Monte Carlo replications.

When $p \in \{1, 3\}$, only one exposure affects the mediators; for $p = 7$, two exposures influence them. Thus, when $\eta = 0$ (no unmeasured confounding) and $p \in \{1, 3\}$, there is no hidden confounding in the outcome model, even when analyzing individual exposures with methods like HIMA2. However, even in the absence of hidden confounder $\mathbf{H}$ in the outcome model (6), $\mathbf{X}_{-k}$ may function as hidden confounders, with the extent of confounding increasing with the dimensionality p of the exposure variable.

$$Y = X_k \gamma_k + \mathbf{M}^\top \boldsymbol{\beta} + \mathbf{X}_{-k}^\top \boldsymbol{\gamma}_{-k} + \mathbf{H}^\top \boldsymbol{\phi} + \epsilon \tag{6}$$

$$M_l = X_k \boldsymbol{\Theta}_{k,l} + \mathbf{X}_{-k}^\top \boldsymbol{\Theta}_{-k,l} + \mathbf{H}^\top \boldsymbol{\Psi}_{2,\cdot,l} + E_{M,l}, \quad l = 1, \cdots, p. \tag{7}$$

Results are shown in Fig. S2, with Fig. S2a depicting scenarios with fewer exposures. We first examine $p = 1$ (first column of Fig. S2a), which aligns with conditions addressed by methods like HIMA2. Without hidden confounding ($\eta = 0$), other methods effectively control the FDR, achieving near-unity power. We adjusted HDMA p-values for multiple comparisons, explaining slight discrepancies from HIMA2 simulation results. As η increases, FDR rises and power declines, although HIMA2 retains relatively higher power.

Next, for $p = 3$ (middle column of Fig. S2a), only one exposure affects the mediator and outcome. Initially, with no hidden confounder ($\eta = 0$), methods like HIMA2 control the FDR only if exposures are independent ($\rho = 0$), yielding near-unity power. Increased correlation among exposures (even at

(a) Few exposures ($p \in \{1, 3, 5\}$). **(b)** Many exposures ($p \in \{20, 50, 100\}$).

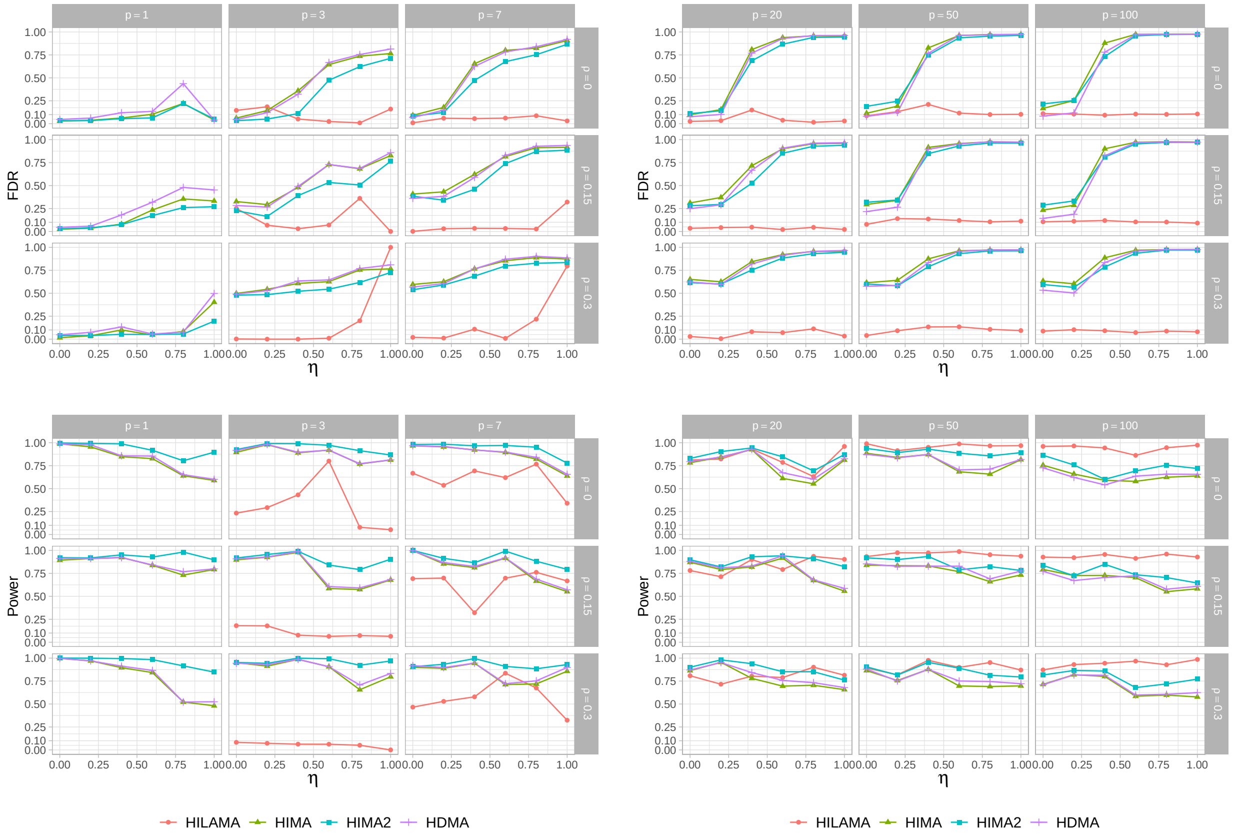

Fig. S2. Comparison results of Empirical False Discovery Rate (FDR) and Empirical Power of different methods. p denotes the dimension of the exposures, ρ denotes the correlation size among the exposures and η denotes the magnitude of hidden confounding. All results are averaged over 100 replications at the nominal FDR level of 0.1.

$\rho = 0.15$) complicates FDR control, leading to higher FDR, similar to issues with latent confounders. When hidden confounders are present, FDR increases with η. For $p = 7$, where two features affect the mediator, similar trends are observed. Specifically, with few exposures, HILAMA underperforms, failing to control the FDR and showing lower power compared to other methods.

Finally, for $p \in \{20, 50, 100\}$ in Fig. S2b, HIMA2 controls the FDR effectively only when exposures are independent and there is no hidden confounding ($\rho = 0, \eta = 0$). As correlations or hidden confounding increases, FDR rises and power declines. In contrast, HILAMA consistently maintains FDR control and achieves the highest power across these scenarios.

Simulation 3: Results from simulation 3 are shown below:

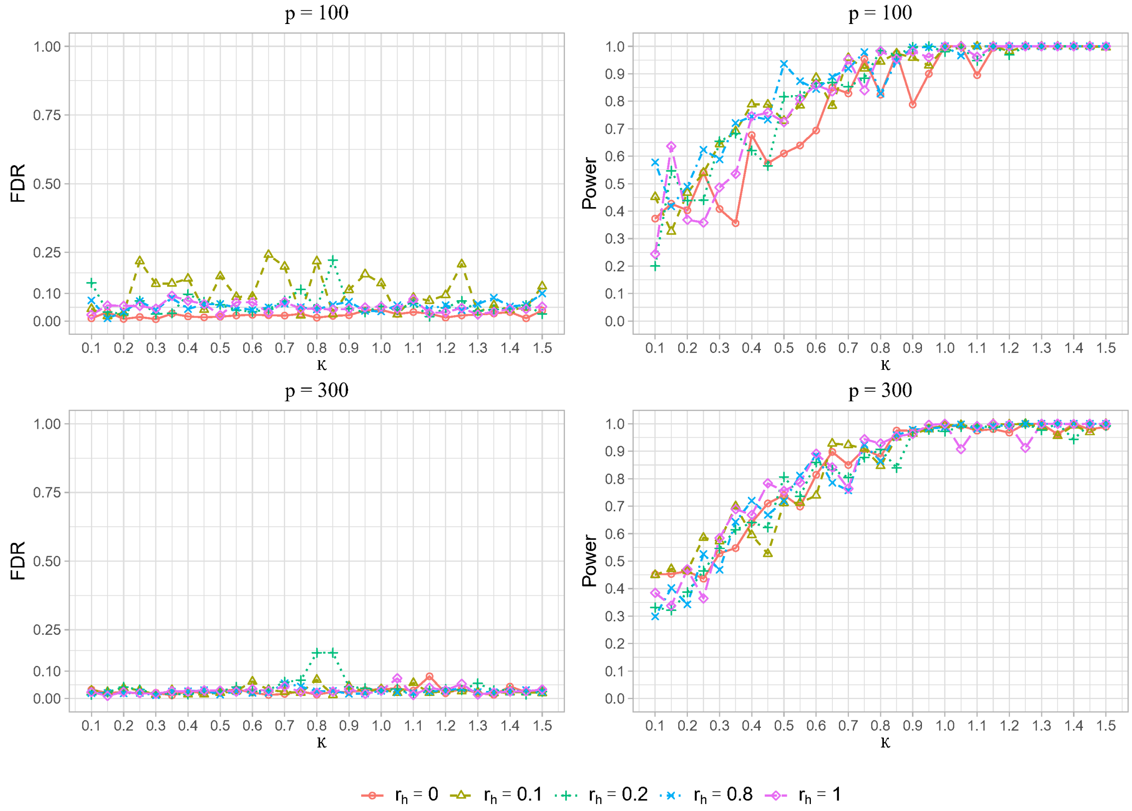

Fig. S3. Comparison results of **simulation 3** with varied signal strength ρ, confounding density r_h and exposure dimension p while fixing $n = 300, q = 100, \kappa = 0.6, \eta = 1, r_p = r_{pq} = 0.1, r = 3, s = 2$. All results are averaged over 100 replications under the nominal FDR level of 0.1.

Application

Singular valued of standardized exposure and mediator data after projection:

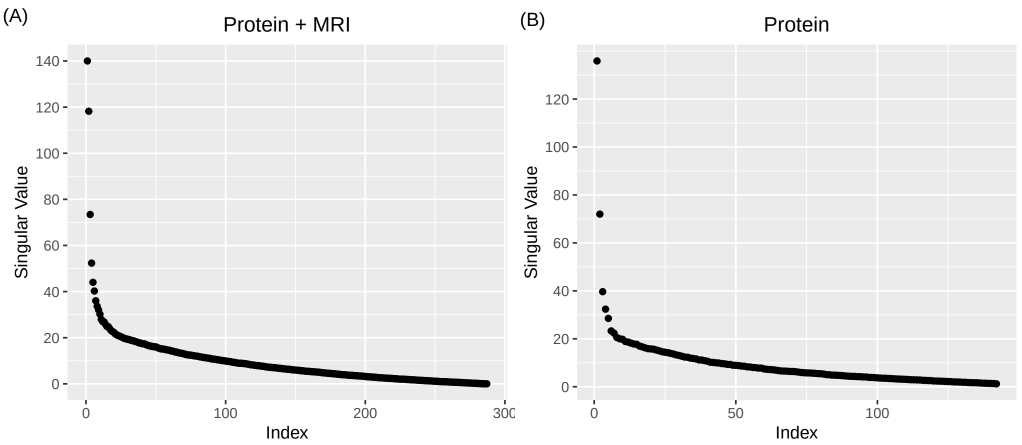

Fig. S4. Singular values of standardized exposure and mediator data after projection. (A) corresponds to the concatenate of protein and MRI data, which is used in the outcome model. (B) corresponds to the protein data, which is used in the mediator model.

Table S1. The summary statistics of the estimated significant paths.

Exposure	Mediator	θ_{ij}	β_j	NIE$_{ij}$	p-value	RID	Region Meaning
NPTXR	R48	0.74	0.22	0.17	0.001	O95502	Left Hippocampus
SE6L1	R48	0.80	0.22	0.18	0.002	Q9BYH1	Left Hippocampus
NPTX2	R48	0.37	0.22	0.08	0.003	P47972	Left Hippocampus
CERU	R48	-0.40	0.22	-0.09	0.004	P00450	Left Hippocampus
SPRL1	R48	-0.48	0.22	-0.11	0.008	Q14515	Left Hippocampus
VGF	R48	-0.60	0.22	-0.13	0.009	O15240	Left Hippocampus
CH3L1	R48	-0.18	0.22	-0.04	0.012	P36222	Left Hippocampus
CERU	R106	-0.48	0.10	-0.05	0.018	P00450	Right angular gyrus
MIME	R106	0.29	0.10	0.03	0.018	P20774	Right angular gyrus
NPTXR	R106	0.53	0.10	0.05	0.018	O95502	Right angular gyrus
SCG3	R48	0.26	0.22	0.06	0.021	Q8WXD2	Left Hippocampus
COCH	R205	-0.20	0.12	-0.02	0.023	O43405	Left triangular part of the inferior frontal gyrus
B3GN1	R48	-0.58	0.22	-0.13	0.024	O43505	Left Hippocampus
NEO1	R48	0.62	0.22	0.14	0.025	Q92859	Left Hippocampus
APOB	R106	0.16	0.10	0.02	0.028	P04114	Right angular gyrus
CERU	R180	-0.36	-0.09	0.03	0.031	P00450	Right planum polare
LPHN1	R180	0.18	-0.09	-0.02	0.031	O94910	Right planum polare
NPTXR	R180	0.54	-0.09	-0.05	0.031	O95502	Right planum polare
APLP2	R180	0.42	-0.09	-0.04	0.032	Q06481	Right planum polare
SCG1	R106	0.20	0.10	0.02	0.033	P05060	Right angular gyrus
CATA	R205	-0.16	0.12	-0.02	0.038	P04040	Left triangular part of the inferior frontal gyrus
PRDX3	R106	-0.14	0.10	-0.01	0.041	P30048	Right angular gyrus
PDYN	R180	-0.22	-0.09	0.02	0.044	P01213	Right planum polare

Table S1 continued from previous page

CERU	R142	-0.44	0.12	-0.05	0.047	P00450	Right middle frontal gyrus
ENOG	R142	-0.16	0.12	-0.02	0.047	P09104	Right middle frontal gyrus
KLK11	R142	-0.14	0.12	-0.02	0.047	Q9UBX7	Right middle frontal gyrus
MOG	R142	-0.36	0.12	-0.04	0.047	Q16653	Right middle frontal gyrus
NEO1	R142	0.73	0.12	0.09	0.047	Q92859	Right middle frontal gyrus
NPTXR	R142	0.73	0.12	0.09	0.047	O95502	Right middle frontal gyrus
BASP1	R205	0.14	0.12	0.02	0.048	P80723	Left triangular part of the inferior frontal gyrus

References

1. Rubin D. Estimating causal effects of treatments in experimental and observational studies. ETS Research Bulletin Series. 1972;1972(2):i–31.

2. Splawa-Neyman J, Dabrowska DM, Speed TP. On the Application of Probability Theory to Agricultural Experiments. Essay on Principles. Section 9. Statistical Science. 1990;5(4):465 – 472. doi:10.1214/ss/1177012031.

3. VanderWeele T, Vansteelandt S. Mediation Analysis with Multiple Mediators. Epidemiologic Methods. 2014;2(1):95–115. doi:doi:10.1515/em-2012-0010.
